# Hybrid Ammonium-M(II) Hydrogenophosphite Hydrates as Bioactive Metal Complexes: Synthesis, DFT and Hirshfeld Surface Analysis, and Anticancer and Anti-Inflammatory Activities

**DOI:** 10.3390/ph19081315

**Published:** 2026-08-20

**Authors:** Zineb Ariba, Houda Zentar, Mohammed Zerrouk, Safaa Hidaoui, Mohammed Er-rajy, Meryem Hrimla, Mustapha Ait El Had, Rachid Chahboun, Juan Sainz, Rachid Ouarsal, Mohammed Lachkar, Fernando J. Reyes-Zurita

**Affiliations:** 1 Engineering Laboratory of Organometallic, Molecular Materials, and Environment (LIMOME), Faculty of Sciences, Sidi Mohamed Ben Abdellah University, Fez 30000, Morocco; zineb.ariba@usmba.ac.ma (Z.A.); safaa.hidaoui@usmba.ac.ma (S.H.);; 2 Department of Biochemistry and Molecular Biology I, Faculty of Sciences, University of Granada, 18071 Granada, Spain; zentarhouda@correo.ugr.es (H.Z.); jsainz@ugr.es (J.S.); 3 LIMAS Laboratory, Faculty of Sciences, Sidi Mohamed Ben Abdellah University, Fez 30003, Morocco; 4 Natural Products Chemistry and Smart Technologies Team, Polydisciplinary Faculty of Larache, Abdelmalek Essaâdi University, Larache 92004, Morocco; 5 Laboratory of Drug Sciences, Faculty of Medicine, Pharmacy and Dental Medicine, Sidi Mohamed Ben Abdellah University, Fez 30000, Morocco; mustapha.aitelhad@usmba.ac.ma; 6 Department of Organic Chemistry, Faculty of Sciences, University of Granada, 18071 Granada, Spain; 7 Genomic Oncology Area, GENYO, Centre for Genomics and Oncological Research, Pfizer/University of Granada/Andalusian Regional Government, PTS, 18016 Granada, Spain; 8 CIBER Epidemiology and Public Health, CIBERESP, 28029 Madrid, Spain; 9 Biosanitary Research Institute of Granada, ibs.GRANADA, 18012 Granada, Spain

**Keywords:** hydrogenophosphite complexes, metal-based compounds, density functional theory, Hirshfeld surface, anticancer activity, anti-inflammatory activity, medicinal inorganic chemistry

## Abstract

**Background/Objectives:** Metal-based coordination compounds are attracting attention in medicinal inorganic chemistry because changes in the metal centre can influence their physicochemical properties and biological responses. This study aimed to evaluate two isostructural ammonium-M(II) hydrogenophosphite hydrates, (NH_4_)_2_[Mg(H_2_O)_6_]_3_(HPO_3_)_4_ (complex **1**) and (NH_4_)_2_[Co(H_2_O)_6_]_3_(HPO_3_)_4_ (complex **2**), as bioactive systems with potential anticancer and anti-inflammatory properties. **Methods:** The complexes were synthesized and characterized by X-ray powder diffraction (XRPD), Fourier-transform infrared spectroscopy (FTIR), thermogravimetric analysis (TGA), density functional theory (DFT) calculations, and Hirshfeld surface analysis. Their cytotoxic activity was evaluated against B16-F10, HT29, HepG2, and HL-60 tumour cell lines. For complex **2**, apoptosis, cell-cycle distribution, and mitochondrial membrane potential were analysed by flow cytometry. Nitric oxide production was measured in LPS-stimulated RAW 264.7 macrophages. **Results:** Complex **2** showed the highest cytotoxic activity, particularly against HL-60 leukaemia cells (IC_50_ = 36.98 μg/mL), whereas complex **1** displayed weaker activity. Complex **2** increased apoptotic cell populations, altered cell-cycle distribution, and induced mitochondrial membrane depolarization in HL-60 cells. Both complexes reduced nitric oxide production, with complex **2** showing the strongest effect (IC_50_ NO = 22.46 μg/mL), exceeding that of diclofenac under the experimental conditions, while complex **1** showed comparable activity to the reference drug. DFT descriptors indicated higher electronic reactivity for complex **2**. **Conclusions:** Replacement of Mg(II) by Co(II) enhances the biological activity of this hydrogenophosphite framework. Complex **2** combines cytotoxic activity against HL-60 cells with apoptosis-associated mitochondrial dysfunction and NO-inhibitory activity in activated macrophages, supporting hydrogenophosphite-based metal complexes as bioactive coordination compounds with potential as multifunctional agents.

## 1. Introduction

Over the past two decades, the field of organic–inorganic hybrid materials has expanded remarkably and has become one of the most dynamic and promising areas of research. Although the concept of combining organic and inorganic components may now seem straightforward, the inherent incompatibility between these two realms has driven chemists and physicists to design a wide variety of architectures, some of which remain experimentally challenging to assemble [1,2]. Hybrid framework materials are defined as crystalline solids in which organic and inorganic units coexist within a continuous network; depending on the connectivity, their structures can extend as one-dimensional chains, two-dimensional layers, or three-dimensional frameworks [3]. Hybrid inorganic–organic framework compounds have attracted considerable attention for a wide range of applications, including separation [4], adsorption [5], medicinal uses [6], magnetism [7], drug delivery [8], sensor technology [9], catalysis [10], ion-exchange [11,12], energy storage [13], and anticancer and anti-inflammatory applications [14,15,16,17,18]. Within this broad family, metal-based coordination compounds and related framework materials, including metal–organic frameworks and metal phosphate/phosphite systems, are increasingly relevant to medicinal chemistry because their structural organization, metal identity, and ligand environment can modulate stability, reactivity, biological interactions, and drug-delivery or bioactive properties [6,8,14,15,16,17,18].

Recent studies further support the biomedical and pharmaceutical relevance of these families of compounds. Transition-metal complexes derived from Schiff base ligands have shown that metal coordination can markedly influence biological activity [19], while MOF-based nanosystems have been explored as drug-delivery platforms for combined anticancer strategies [20]. In parallel, structural, DFT, molecular-modelling, DNA-binding, and electrochemical-sensing studies have provided complementary approaches to relate molecular structure, electronic properties, and functional performance to potential biomedical or pharmaceutical applications [21,22].

The majority of the structures reported so far consist of inorganic clusters connected by bicoordinated chalcogen atoms. Organic ligands can also be used to link these clusters, enabling the creation of novel materials with distinct chemical and physical properties. Such hybrid materials exhibit significant optical and photochemical properties due to the synergistic effects of their organic and inorganic components [23,24]. The combination of robust inorganic clusters and versatile organic ligands has attracted considerable attention because of their remarkable structural features and potential applications in various fields, including gas storage [25], luminescence [26], and photocatalysis [27,28] and biomedical applications. Additionally, these materials have been shown to function as corrosion inhibitors, offering an alternative to conventional organic chemicals [29]. The rational design and controlled synthesis of inorganic–organic hybrid materials with a desired structure and prospective use remain a significant challenge, despite the fact that several remarkable structures have been created. Recently, a considerable number of functional inorganic–organic hybrid materials based on metal phosphate and phosphite clusters have been reported [10]. Due to their unique structural characteristics, which range from linear and layered polymers to open-framework assemblies with extra-large ring channels, coordination assemblies based on metal phosphite frameworks represent a well-studied class of materials. The incorporation of metal ions with the three-connected pseudopyramidal HPO_3_ ligand, instead of the more conventional phosphate groups, provides a practical synthetic toolkit for achieving structural diversity that is typically lacking in four-connected (4–c) metal phosphates [30,31,32,33].

From a medicinal inorganic chemistry perspective, such metal phosphite-based systems may provide useful models to examine how changes in the metal centre affect biological activity and cellular behaviour. Although the single-crystal structures of these Mg(II) and Co(II) hydrogenophosphite hydrates were previously reported by our group [34,35], their comparative physicochemical, electronic, supramolecular, anticancer, and anti-inflammatory profiles have not been investigated in an integrated pharmaceutical context. The compounds (NH_4_)_2_[Mg(H_2_O)_6_]_3_(HPO_3_)_4_ (**1**) and (NH_4_)_2_[Co(H_2_O)_6_]_3_(HPO_3_)_4_ (**2**) belong to a relatively unexplored family of metal-phosphite coordination materials. To the best of our knowledge, the reported ammonium-M(II) hydrogenophosphite hydrate family comprises three reported complexes containing Mg, Co and Ni. No biological studies on the Ni complex have been reported to date. Given their interesting biological activities, these materials may represent an emerging class of bioactive metal-phosphite materials rather than analogues of existing phosphorus-containing drugs.

In this work, we report the synthesis in powder form of two complexes, (NH_4_)_2_[Mg(H_2_O)_6_]_3_(HPO_3_)_4_ (complex **1**) and (NH_4_)_2_[Co(H_2_O)_6_]_3_(HPO_3_)_4_ (complex **2**), and evaluate how replacement of Mg(II) by Co(II) affects their structural, electronic, and biological activity and cellular response. To assess molecular reactivity and provide detailed insights into electronic behaviour at the molecular level, density functional theory (DFT) was used. The calculations were carried out within the framework of the generalized gradient approximation (GGA) with the double numerical plus polarization (DNP) basis set, as implemented in the DMol3 computational package. To elucidate the nature of intermolecular interactions in the reported phosphite compounds, Hirshfeld surface (HS) analysis was performed. In addition, 2D fingerprint plots (FPs) were generated using the CrystalExplorer program to provide a quantitative visualization of intermolecular contacts. The in vitro anticancer activities of both complexes were evaluated against four tumour cell lines: B16-F10 (melanoma), HT29 (colorectal adenocarcinoma), HepG2 (hepatocellular carcinoma) and HL-60 (acute myeloid leukaemia). For the most active compound, complex **2**, apoptosis induction, cell-cycle distribution, and mitochondrial membrane potential were analysed by flow cytometry. The anti-inflammatory response of each complex was also evaluated by analysing nitrite levels in LPS-activated RAW 264.7 macrophages.

## 2. Results and Discussion

### 2.1. Chemistry

#### 2.1.1. Structural Description

**Figure 1:**
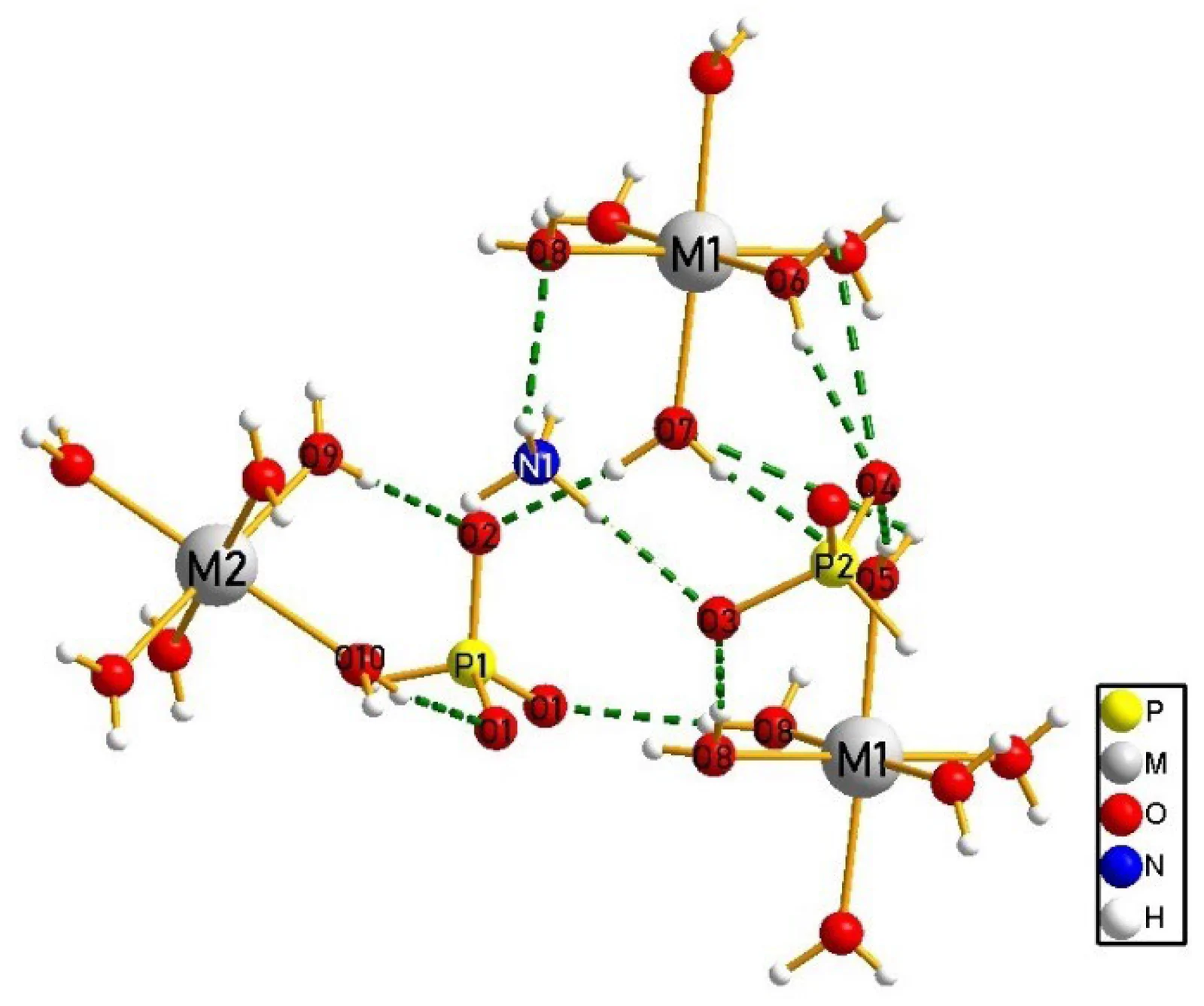
Intermolecular N–H···O hydrogen bonds in (NH_4_)_2_[M(H_2_O)_6_]_3_(HPO_3_)_4_ [M = Co, Mg] are represented as dashed green lines.

**Figure 2:**
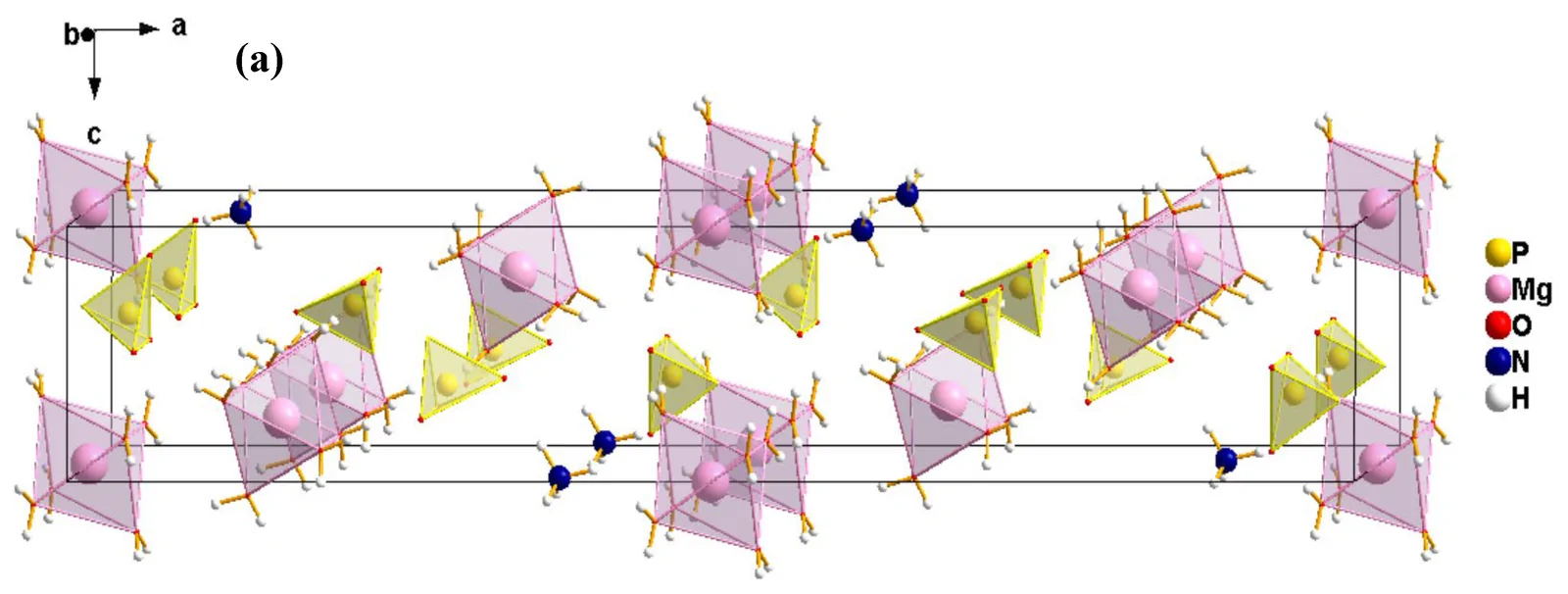

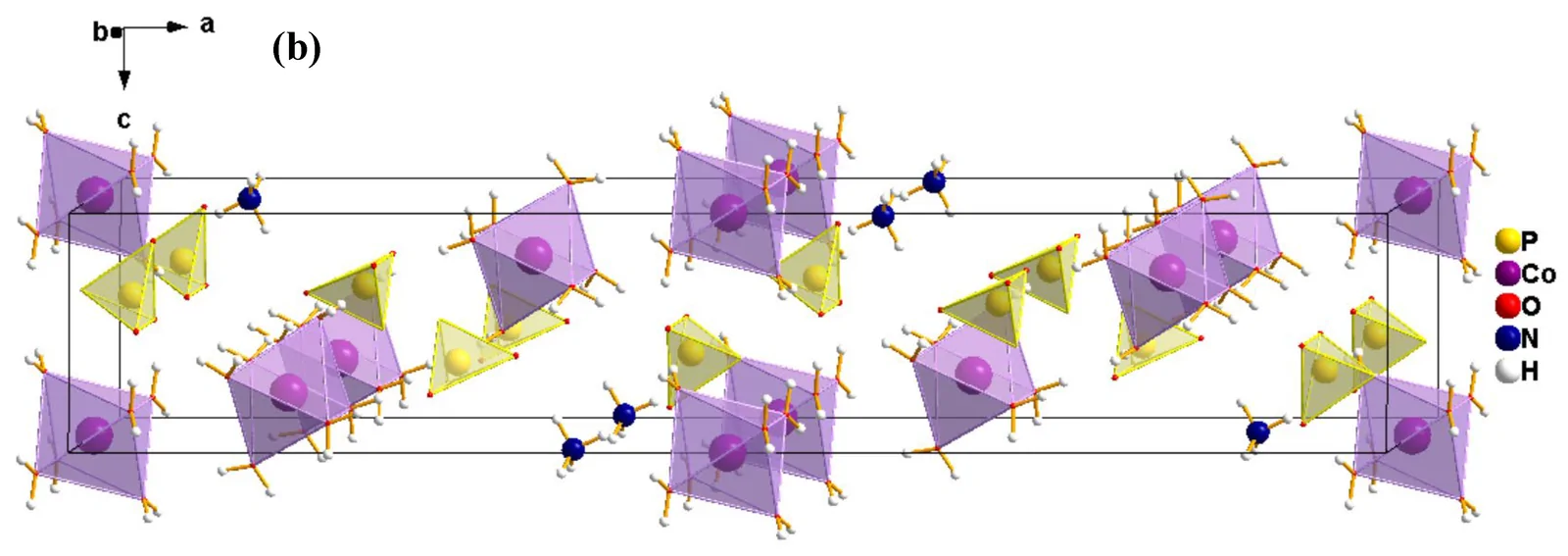
(**a**) A projection along b-axis of (NH_4_)_2_[Mg(H_2_O)_6_]_3_(HPO_3_)_4_ and (**b**) (NH_4_)_2_[Co(H_2_O)_6_]_3_(HPO_3_)_4_.

A perspective view of the structural units of (NH_4_)_2_[M(H_2_O)_6_]_3_(HPO_3_)_4_ (M = Mg, Co) is shown in Figure 1, illustrating the coordination of nitrogen, oxygen, and hydrogen atoms with the phosphorus and divalent metal M atoms. The crystal structure of the phosphites (NH_4_)_2_[M(H_2_O)_6_]_3_(HPO_3_)_4_ (M = Mg, Co) can be described in terms of three distinct units: [M(H_2_O)_6_]^2+^ (M = Mg, Co), [HPO_3_]^2–^ and [NH_4_]^+^. These units are connected by a complex network of hydrogen bonds, comprising two different kinds of hydrogen bonds, as shown in Figure 1: N–H···O and O–H···O (Table 1 and Table 2). This hydrogen-bonding network links the discrete ionic units and contributes to the cohesion of the supramolecular framework. Projections of the crystal structures are illustrated in Figure 2a,b, where the three polyhedra M(H_2_O)_6_, HPO_3_, and NH_4_ extend along the (001) plane. The symmetry sites of the two independent divalent cations, M(1) and M(2), are m and 2/m, respectively. Each cation is surrounded by six oxygen atoms, each from a water molecule. The average M–O bond distances in (NH_4_)_2_[M(H_2_O)_6_]_3_(HPO_3_)_4_ [M = Co and Mg] are 2.093(2) Å and 2.072(2) Å, respectively. The M(H_2_O)_6_ polyhedra are isolated in each structure, with the shortest M···M distances being 6.187 Å for M = Co and 6.166 Å for M = Mg, respectively [34,35].

#### 2.1.2. Powder X Ray Diffraction

**Figure 3:**
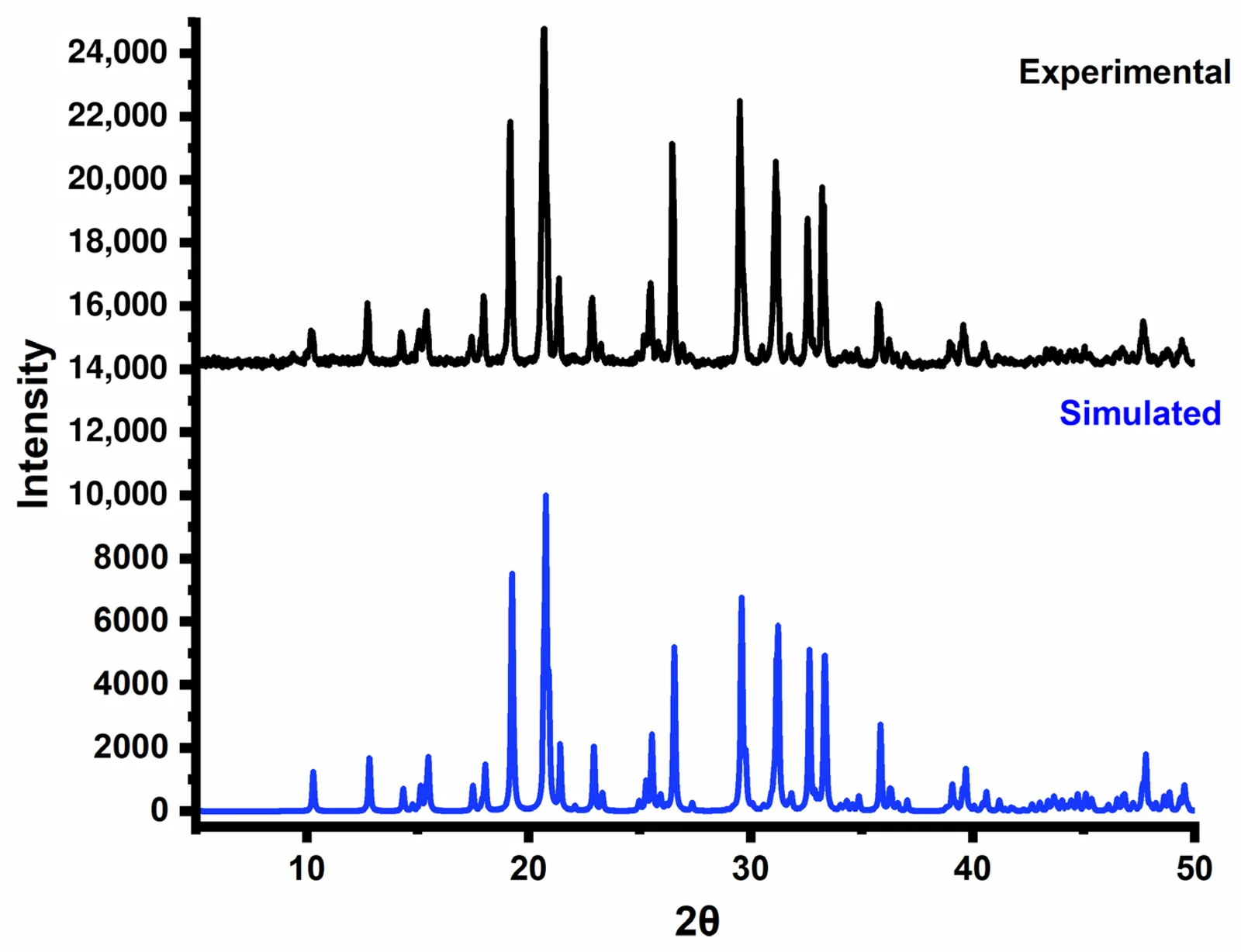
Experimental (black line) and simulated (blue line) X-ray diffraction patterns of (NH_4_)_2_[Mg(H_2_O)_6_]_3_(HPO_3_)_4_.

Prior to further characterization and biological evaluation, powder X-ray diffraction analysis was performed on the powder samples used in this study (Figure 3). The resulting XRPD patterns were consistent with the theoretically simulated patterns generated from single-crystal X-ray diffraction (SXRD) data, thus confirming the crystalline phase purity and homogeneity of the synthesized complexes.

#### 2.1.3. Infrared Spectroscopy

**Figure 4:**
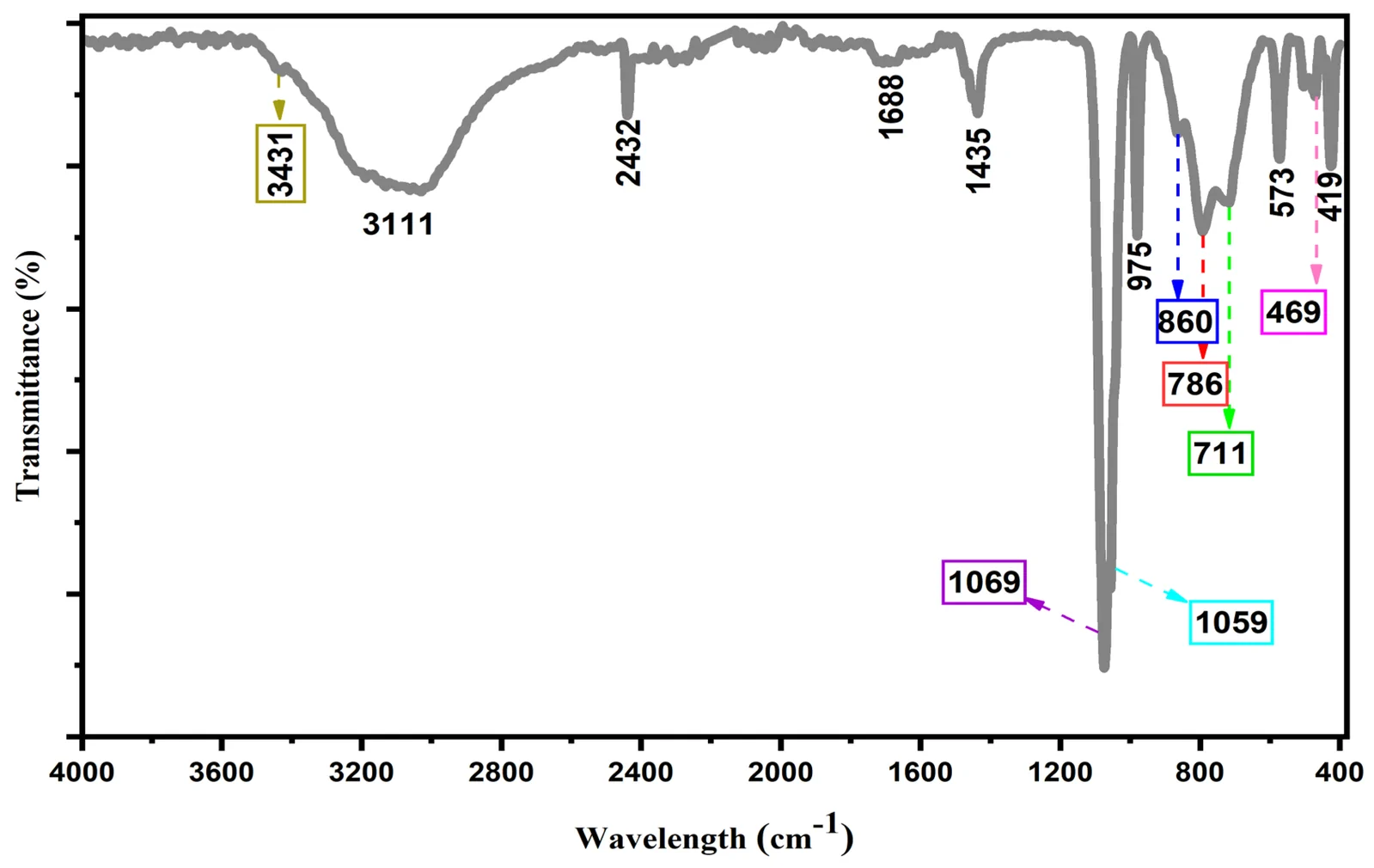
Infrared spectrum of (NH_4_)_2_[Mg(H_2_O)_6_]_3_(HPO_3_)_4_. Coloured arrows and boxes indicate the labelled absorption bands, whose assignments are provided in Table 3.

**Figure 5:**
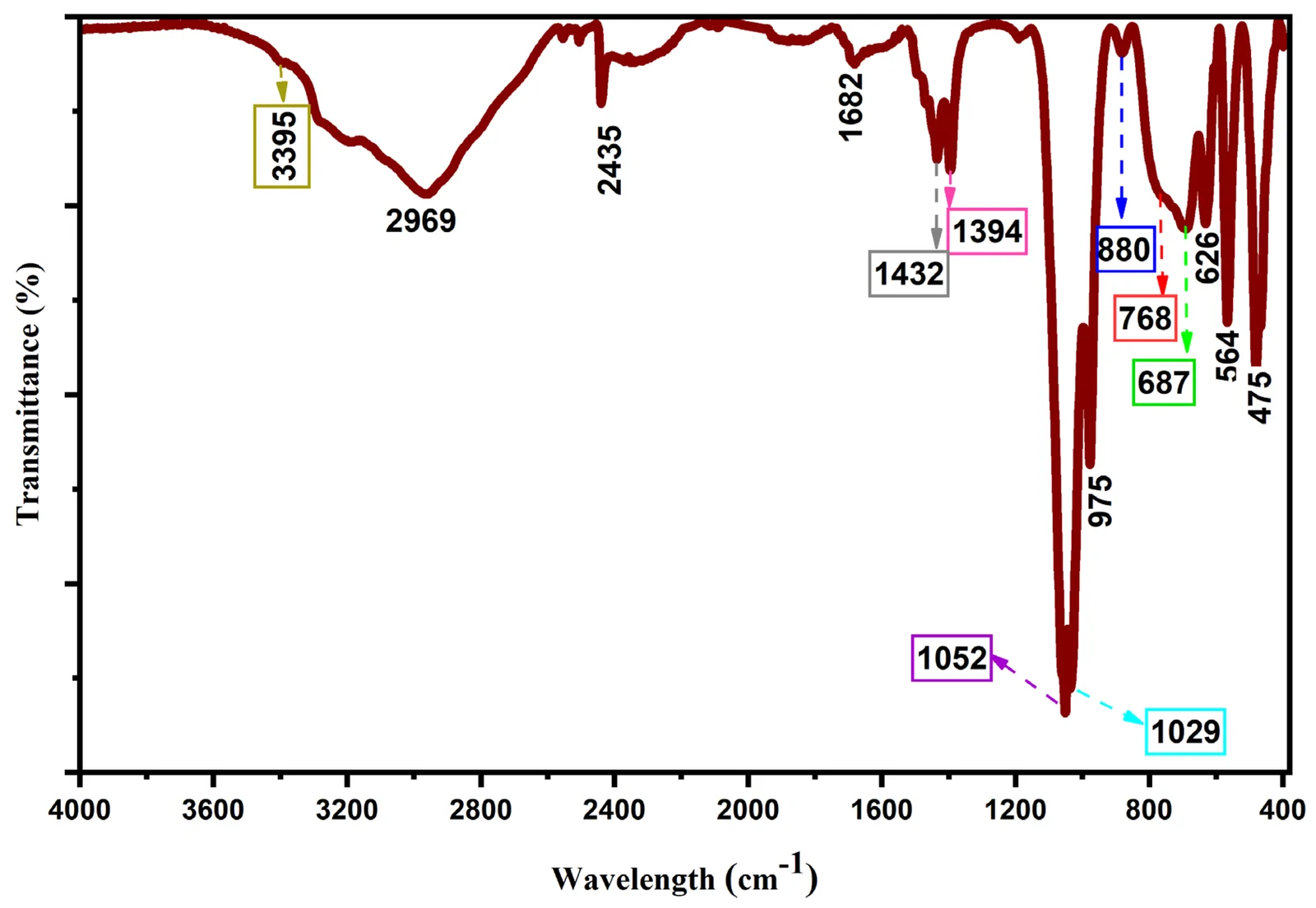
Infrared spectrum of (NH_4_)_2_[Co(H_2_O)_6_]_3_(HPO_3_)_4_. Coloured arrows and boxes indicate the labelled absorption bands, whose assignments are provided in Table 3.

Following the structural and crystallographic description of the isostructural materials (NH_4_)_2_[M(H_2_O)_6_]_3_(HPO_3_)_4_ [M = Mg and Co], further structural insights were obtained through infrared (IR) vibrational spectroscopy. The IR spectra of the complexes are shown in Figure 4 and Figure 5, and the corresponding bands are summarized in Table 3. The vibrational modes of the PO_3_, P–H, NH_4_, and H_2_O groups were assigned by interpreting the infrared spectra. The bands in the region 3440–2960 cm^−1^ correspond to the stretching vibrations of the water molecule and the NH_4_^+^ ion [36]. In phosphate complexes, the bands around 3400 cm^−1^ are attributed to the stretching vibrations of the OH group in the H_2_O molecule [37]. The vibration band of the water molecule appears at around 800 cm^−1^. The bands observed in the region 2960–2850 cm^−1^ correspond to the combination vibration modes of the NH_4_^+^ ion. The deformation bands are assigned to the NH_4_^+^ ion [δ(NH_4_)] in the range of 1430–1440 cm^−1^ and to the water molecule [δ(H_2_O)] in the range of 1690–1680 cm^−1^ [38,39]. The stretching vibration bands of the P–H bond (υ(P–H)) are observed in the range of 2432–2435 cm^−1^, whereas the deformation vibration bands (δ(P–H)) are detected between 1040 and 979 cm^−1^ [40]. The P=O bond vibrations are observed in the range of 1069–1052 cm^−1^, while the symmetric and asymmetric deformation vibrations of the PO_3_ groups are responsible for the bands appearing in the 460–630 cm^−1^ region [41].

### 2.2. Thermal Behaviour

**Figure 6:**
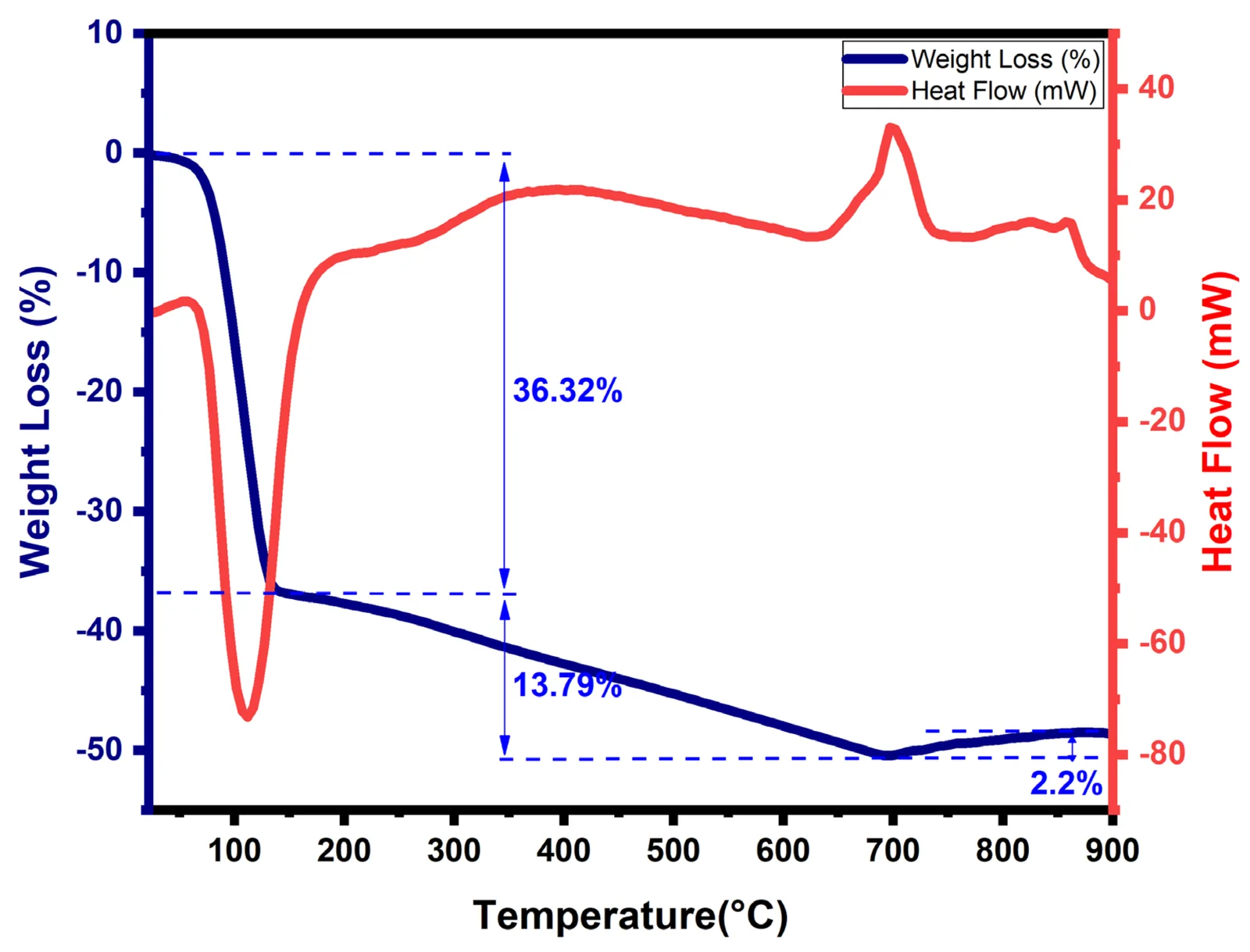
TGA-DTA analyses of (NH_4_)_2_[Mg(H_2_O)_6_]_3_(HPO_3_)_4_. Dashed lines and arrows indicate the mass-loss steps and their corresponding percentage losses; the curve colours are identified in the figure legend.

For the complex (NH_4_)_2_[Mg(H_2_O)_6_]_3_(HPO_3_)_4_, the dehydration occurring in the 67–140 °C temperature range takes place in two steps, with the release of 15 water molecules, corresponding to a total mass loss of 36.32% of the initial sample, as shown in Figure 6. This dehydration is also confirmed by DTA, where a pronounced endothermic peak is observed at ≈112 °C [42]. The mass loss of 13.79% in the temperature range of 170–700 °C corresponds to the release of two ammonia molecules (NH_3_) and four water molecules. However, three water molecules are crystallographically equivalent, while the fourth originates from the condensed phosphite, where the condensation of phosphite groups (HPO_3_^2−^) leads to the formation of magnesium polyphosphates, namely metaphosphate Mg(PO_3_)_2_ and diphosphate Mg_2_(P_2_O_7_), which are stabilized through coordination with magnesium ions (Mg^2+^) [43,44,45].

**Figure 7:**
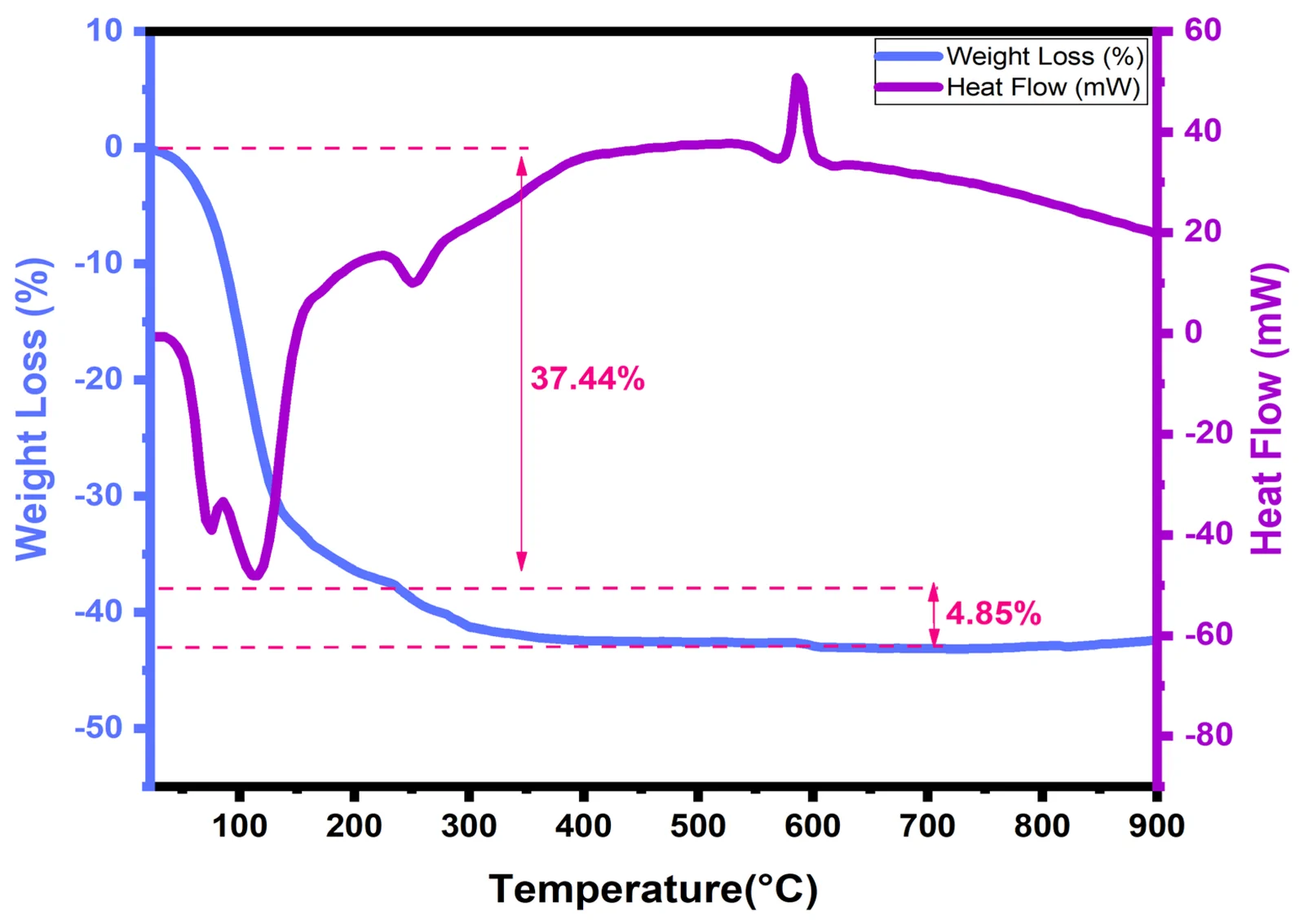
TGA-DTA analyses of (NH_4_)_2_[Co(H_2_O)_6_]_3_(HPO_3_)_4_. Dashed lines and arrows indicate the mass-loss steps and their corresponding percentage losses; the curve colours are identified in the figure legend.

For the complex (NH_4_)_2_[Co(H_2_O)_6_]_3_(HPO_3_)_4_, a continuous weight loss step is also observed in the temperature range from 200 to 600 °C, as illustrated in Figure 7. This weight loss is attributed to a complex chemical reaction. In the first step, occurring between 28 and 235 °C, the coordinated water molecules are released from (NH_4_)_2_[Co(H_2_O)_6_]_3_(HPO_3_)_4_. Subsequently, ammonia is eliminated through the decomposition of the ammonium ion, while its proton is transferred to the phosphite groups, leading to their conversion into phosphorous acid. A self-oxidation–reduction reaction of phosphorous acid may occur above 235 °C, leading to the formation of phosphine (PH_3_) and phosphoric acid (H_3_PO_4_) [43].

The high-temperature thermal event observed at approximately 700 °C for complex **1** and 600 °C for complex **2** is tentatively attributed to a solid-state transformation of the final decomposition products. Following dehydration and ammonium elimination, intermediate phosphorus-containing phases are expected to form and subsequently undergo oxidation and structural reorganization towards thermodynamically more stable metal phosphate phases under air atmosphere. Therefore, the exothermic event observed at approximately 700 °C for complex **1** and 600 °C for complex **2** can be associated with the reorganization of the metal polyphosphate residue, mainly involving Mg(PO_3_)_2_ and diphosphate Mg_2_(P_2_O_7_) for complex **1**, and Co(PO_3_)_2_ and Co_2_P_2_O_7_ phases for complex **2**. It is assumed that the PO_3_ groups originate from the phosphite units present in the complex, whereas the P_2_O_7_ groups are formed through subsequent complex chemical reactions.

The lower transformation temperature observed for the cobalt complex **2** compared with its magnesium analogue may be related to the different thermal behaviour and higher reactivity of cobalt-containing intermediates and to a lower activation energy for the phase transformation process. In the absence of high-temperature powder X-ray diffraction analysis of the decomposition residues, the exact nature and crystal structure of the final phases cannot be established unambiguously, and this assignment should therefore be regarded as tentative.

### 2.3. Computational Results

#### 2.3.1. Chemical Reactivity

**Figure 8:**
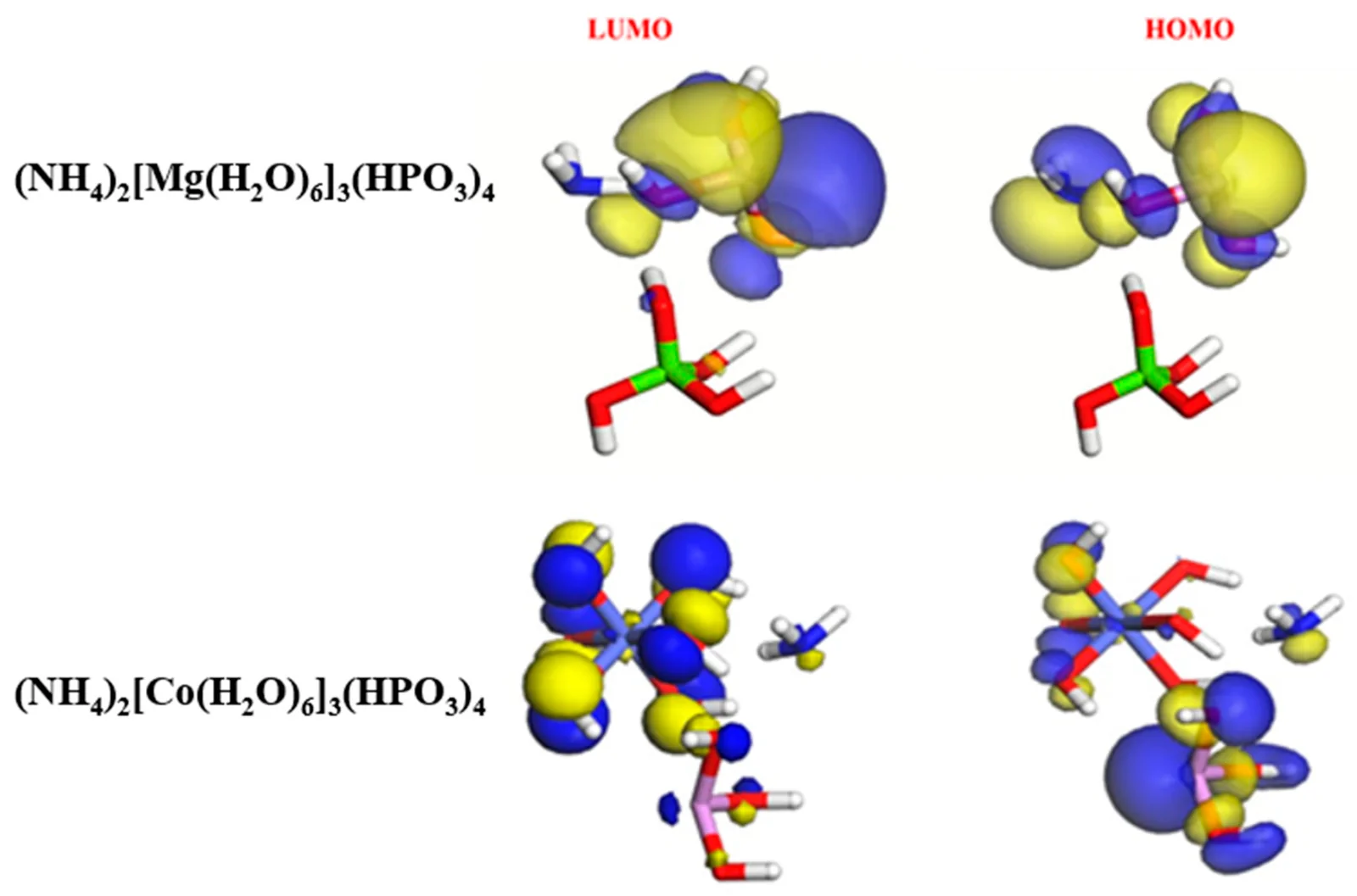
The HOMO and LUMO of the asymmetric molecules (NH_4_)_2_[Mg(H_2_O)_6_]_3_(HPO_3_)_4_ and (NH_4_)_2_[Co(H_2_O)_6_]_3_(HPO_3_)_4_ were calculated at the DFT/GGA level. Yellow and blue surfaces represent opposite phases of the molecular orbitals.

In recent years, considerable attention has been given to the discovery of novel, effective, and biocompatible compounds with potential anticancer and anti-inflammatory activities, using cost-effective computational approaches as complements to conventional experimental investigations [46]. In this section, we use quantum mechanical calculations to explore the reactivity of (NH_4_)_2_[Mg(H_2_O)_6_]_3_(HPO_3_)_4_ and (NH_4_)_2_[Co(H_2_O)_6_]_3_(HPO_3_)_4_ molecules in isolation, focusing on their electronic structures. These calculations were intended to compare the intrinsic electronic properties of the Mg(II) and Co(II) complexes and should be interpreted as descriptors of relative molecular reactivity rather than as direct evidence of binding to specific biological targets. Figure 8 shows the HOMO and LUMO iso-surfaces for the two phosphites (NH_4_)_2_[Mg(H_2_O)_6_]_3_(HPO_3_)_4_ and (NH_4_)_2_[Co(H_2_O)_6_]_3_(HPO_3_)_4_, as determined by DFT calculations. These iso-surfaces illustrate the spatial distribution of electron density, highlighting regions of high electron-donor and electron-acceptor affinities. The distributions of HOMO and LUMO offer valuable insights into the electronic characteristics of the compounds, which are relevant to understanding relative chemical reactivity and functional behaviour.

Figure 8 shows that the iso-surface HOMO densities for both (NH_4_)_2_[Mg(H_2_O)_6_]_3_(HPO_3_)_4_ and (NH_4_)_2_[Co(H_2_O)_6_]_3_(HPO_3_)_4_ are primarily concentrated around the HPO_3_ groups. This indicates that these sites may contribute to the electron-donating character of the compounds, which may be relevant to their relative chemical reactivity. Similarly, the LUMO iso-surface distributions of both compounds are mainly localized around the HPO_3_ and Co(H_2_O) groups, respectively, indicating that these regions are more favourable for accepting electrons from electron-donating species. These electronic characteristics identify possible electron-donor and electron-acceptor regions within the complexes and provide a basis for comparing their intrinsic reactivity. The HOMO and LUMO energy values, together with the energy gap (ΔE), provide important information about the electronic properties and chemical reactivity of the molecules [47]. As presented in Table 4, the calculated energy gaps for (NH_4_)_2_[Mg(H_2_O)_6_]_3_(HPO_3_)_4_ and (NH_4_)_2_[Co(H_2_O)_6_]_3_(HPO_3_)_4_ are 2.207 and 0.134 eV, respectively. A larger energy gap is generally associated with higher molecular stability and lower chemical reactivity, whereas a smaller energy gap reflects greater electronic mobility and may favour molecular interactions. Therefore, the lower energy gap observed for the cobalt-based compound is consistent with increased electronic reactivity and may be related to its stronger biological profile. Consequently, (NH_4_)_2_[Co(H_2_O)_6_]_3_(HPO_3_)_4_ is predicted to exhibit greater reactivity compared to (NH_4_)_2_[Mg(H_2_O)_6_]_3_(HPO_3_)_4_, making it more prone to chemical interactions. Additional electronic properties of these molecules are also summarized in Table 4.

#### 2.3.2. Intermolecular Interactions Calculations

**Figure 9:**
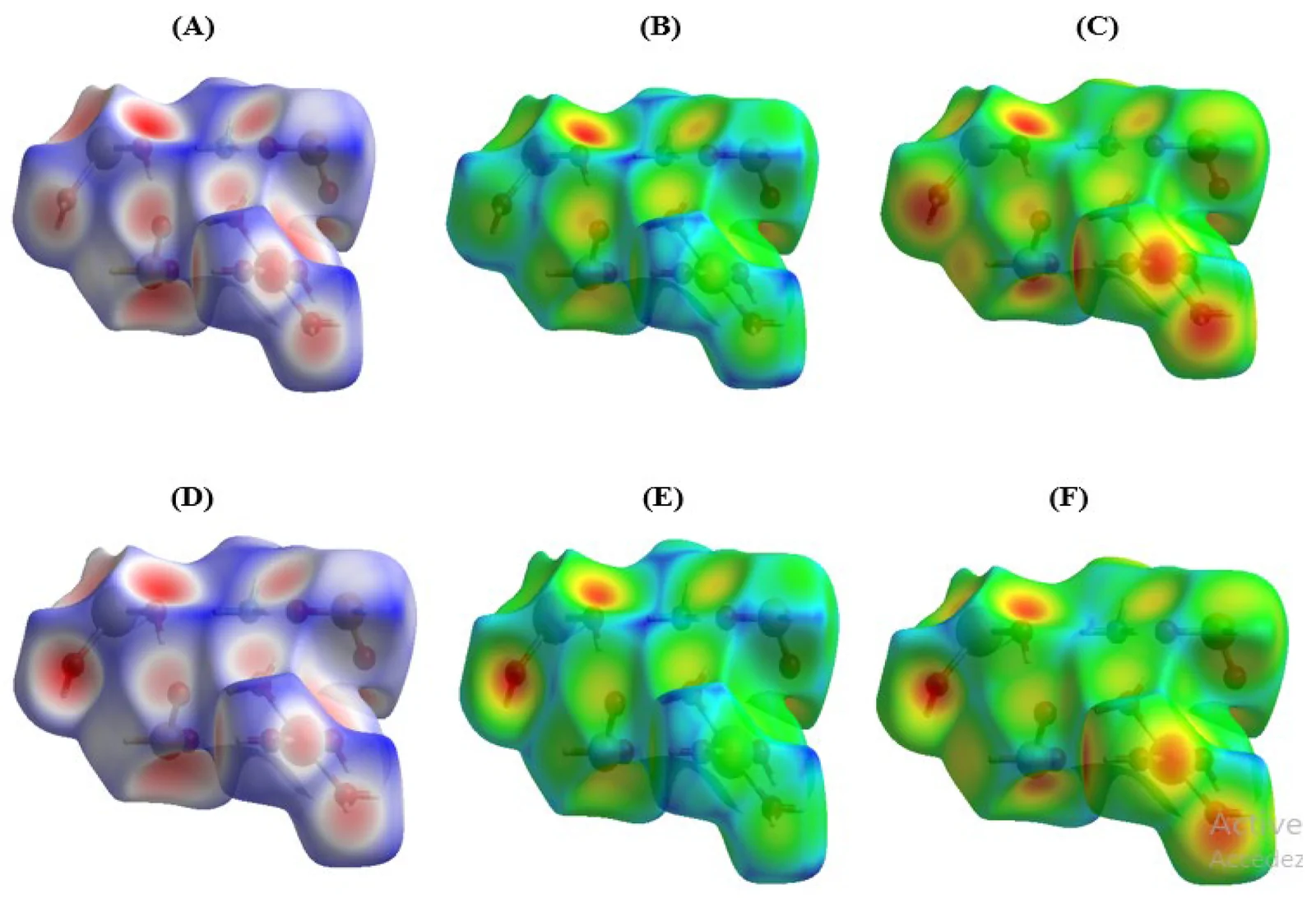
Hirshfeld surfaces mapped with *d_norm_* (**A**), *de* (**B**), and *di* (**C**) for (NH_4_)_2_[Co(H_2_O)_6_]_3_(HPO_3_)_4_; *d_norm_* (**D**), *de* (**E**), and *di* (**F**) for (NH_4_)_2_[Mg(H_2_O)_6_]_3_(HPO_3_)_4_. For the *d_norm_* surfaces, red and blue regions indicate contacts shorter and longer than the sum of the van der Waals radii, respectively; the colours in the *de* and *di* surfaces represent the corresponding external and internal contact distances.

The Hirshfeld surface (HS) is an effective method for describing the surface properties of molecules. The 3D Hirshfeld surfaces of the studied phosphite complexes, depicted in Figure 9A–F, show surfaces mapped over *d_norm_*, *d_e_*, and *d_i_*. In these maps, *d_norm_* highlights contacts that are shorter or longer than the sum of the corresponding van der Waals radii, whereas *d_e_* and *d_i_* describe the distances from a point on the surface to the nearest external and internal atoms, respectively. In the standard *d_norm_* surfaces (Figure 9A–D), the red spots are attributed to the O–H∙∙∙O and N–H∙∙∙O hydrogen bonds, which represent the closest intermolecular contacts in the crystal structures [41,48,49]. A few orange and yellow spots are also observed on the *d_norm_* surface, resulting from weak H∙∙∙H contacts (Figure 9C–F).

**Figure 10:**
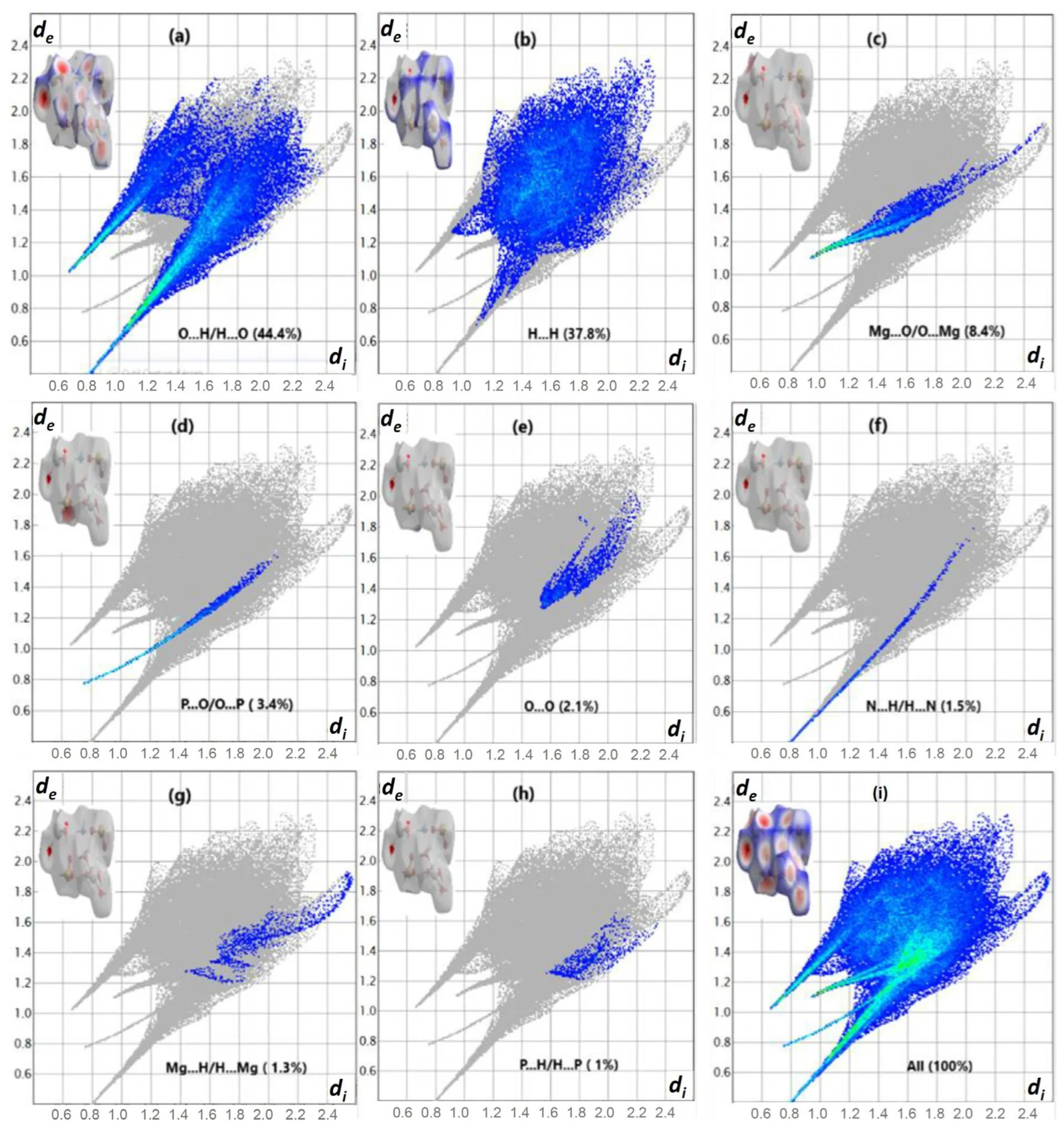
Full 2D fingerprint plot for (NH_4_)_2_[Mg(H_2_O)_6_]_3_(HPO_3_)_4_, showing all interactions. The axes represent *de* and *di*, the distances from the Hirshfeld surface to the nearest external and internal atoms, respectively; grey points represent the complete fingerprint background, whereas coloured points indicate the selected contact contribution. Sub-plots: (**a**) O∙∙∙H/H∙∙∙O, (**b**) H∙∙∙H, (**c**) Mg∙∙∙O/O∙∙∙Mg, (**d**) P∙∙∙O/O∙∙∙P, (**e**) O∙∙∙O, (**f**) N∙∙∙H/H∙∙∙N, (**g**) Mg∙∙∙H/H∙∙∙Mg, (**h**) P∙∙∙H/H∙∙∙P, (**i**) all contacts.

**Figure 11:**
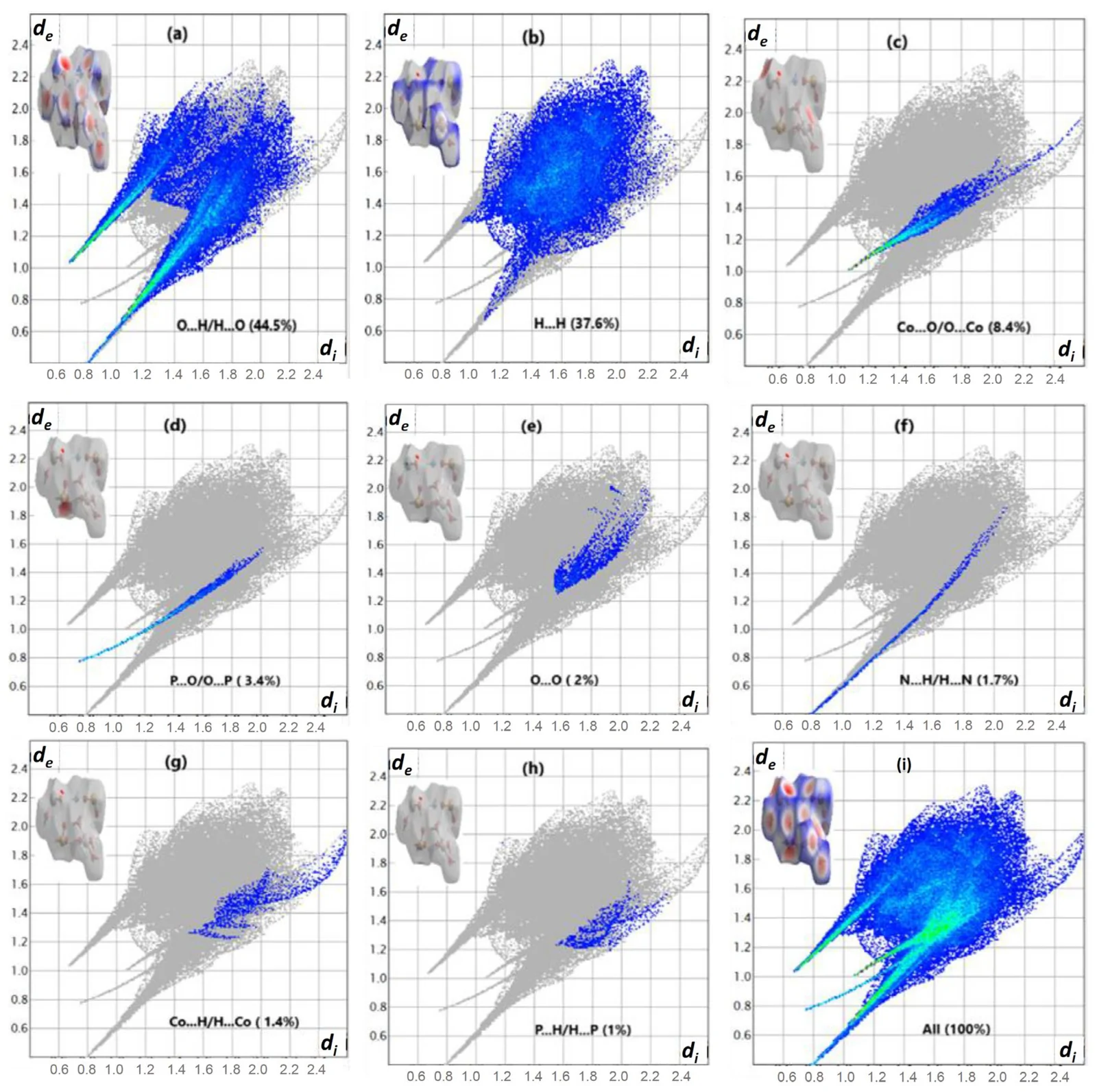
Full 2D fingerprint plot for (NH_4_)_2_[Co(H_2_O)_6_]_3_(HPO_3_)_4_, showing all interactions. The axes represent *de* and *di*, the distances from the Hirshfeld surface to the nearest external and internal atoms, respectively; grey points represent the complete fingerprint background, whereas coloured points indicate the selected contact contribution. Sub-plots: (**a**) O∙∙∙H/H∙∙∙O, (**b**) H∙∙∙H, (**c**) Co∙∙∙O/O∙∙∙Co, (**d**) P∙∙∙O/O∙∙∙P, (**e**) O∙∙∙O, (**f**) N∙∙∙H/H∙∙∙N, (**g**) Co∙∙∙H/H∙∙∙Co, (**h**) P∙∙∙H/H∙∙∙P, (**i**) all contacts.

The 2D fingerprint plots were generated to obtain quantitative information on the individual contributions of all interactions in the crystal structures. Fingerprint plots for complexes 1 and 2 are shown in Figure 10 and Figure 11, respectively. In complex **1**, the most significant intermolecular contacts were O∙∙∙H/H∙∙∙O (44.4%, Figure 10a) and H∙∙∙H (37.8%, Figure 10b). The O∙∙∙H/H∙∙∙O interactions appear as two sharp spikes in the 2D fingerprint plot, characteristic of short hydrogen-bonding interactions, highlighting their major role in the stability and cohesion of the crystal packing. The second most significant contribution arises from H∙∙∙H contacts, which result from the abundance of hydrogen atoms on the molecular surface. These contacts appear as a broad central region of scattered points in the 2D fingerprint plot, reflecting numerous weak van der Waals interactions. Other interactions in complex **1** include Mg∙∙∙O/O∙∙∙Mg (8.4%, Figure 10c), which appear as a sharp spike in the lower region of the fingerprint map, with the shortest (*di* + *d_e_*) contact distance of approximately 2.1 Å, followed by P∙∙∙O/O∙∙∙P (3.4%, Figure 10d), O···O (2.1%, Figure 10e), N∙∙∙H/H∙∙∙N (1.5%, Figure 10f), Mg∙∙∙H/H∙∙∙Mg (1.3%, Figure 10g), and P∙∙∙H/H∙∙∙P (1.0%, Figure 10h), all of which make only minor contributions to the Hirshfeld surface.

A very similar distribution of intermolecular contacts was observed for complex **2**; the dominant interactions were O∙∙∙H/H∙∙∙O (44.5%, Figure 11a) and H∙∙∙H (37.6%, Figure 11b), confirming that hydrogen bonding interactions govern the crystal packing. The Co∙∙∙O/O∙∙∙Co contacts give rise to a comparable sharp spike, with the shortest contact distance at (*d_i_* + *d_e_*) ≈ 2.09 Å, and likewise account for 8.4% of the Hirshfeld surface (Figure 11c). Minor contributions arise from P∙∙∙O/O∙∙∙P (3.4%, Figure 11d), O∙∙∙O (2%, Figure 11e), N∙∙∙H/H∙∙∙N (1.7%, Figure 11f), Co∙∙∙H/H∙∙∙Co (1.4%, Figure 11g), and P∙∙∙H/H∙∙∙P (1%, Figure 11h). These results reveal that replacing Mg(II) with Co(II) has little effect on the overall intermolecular contact distribution within the crystal structures.

### 2.4. Biological Activities

#### 2.4.1. Anti-Cancer Activity

Cytotoxicity in cancer cell lines. Cell viability assays are essential for evaluating the cytotoxic potential of investigational complexes against cancer cell lines. In this study, the antiproliferative effects of complexes **1** and **2** were evaluated in vitro using the MTT (3-(4,5-dimethylthiazol-2-yl)-2,5-diphenyltetrazolium bromide) colorimetric assay on B16-F10 (murine melanoma), HT29 (colorectal adenocarcinoma), and HepG2 (hepatocellular carcinoma) cell lines. Cytotoxicity against the HL-60 human acute promyelocytic leukaemia (APL) cell line was determined using the resazurin reduction assay. Both methodologies are widely recognized for quantifying metabolic activity as a proxy for cell viability. The complexes were tested at serial concentrations for 72 h, and the half-maximal inhibitory concentration (IC_50_) values were estimated for each complex (Table 5). Additionally, IC_20_ and IC_80_ values were calculated to delineate the range of cytotoxicity. All values are presented as mean ± standard deviation (SD) from at least two independent experiments performed in triplicate. The growth-inhibitory activity of complexes **1** and **2** after 72 h of exposure is summarized in Table 5.

**Figure 12:**
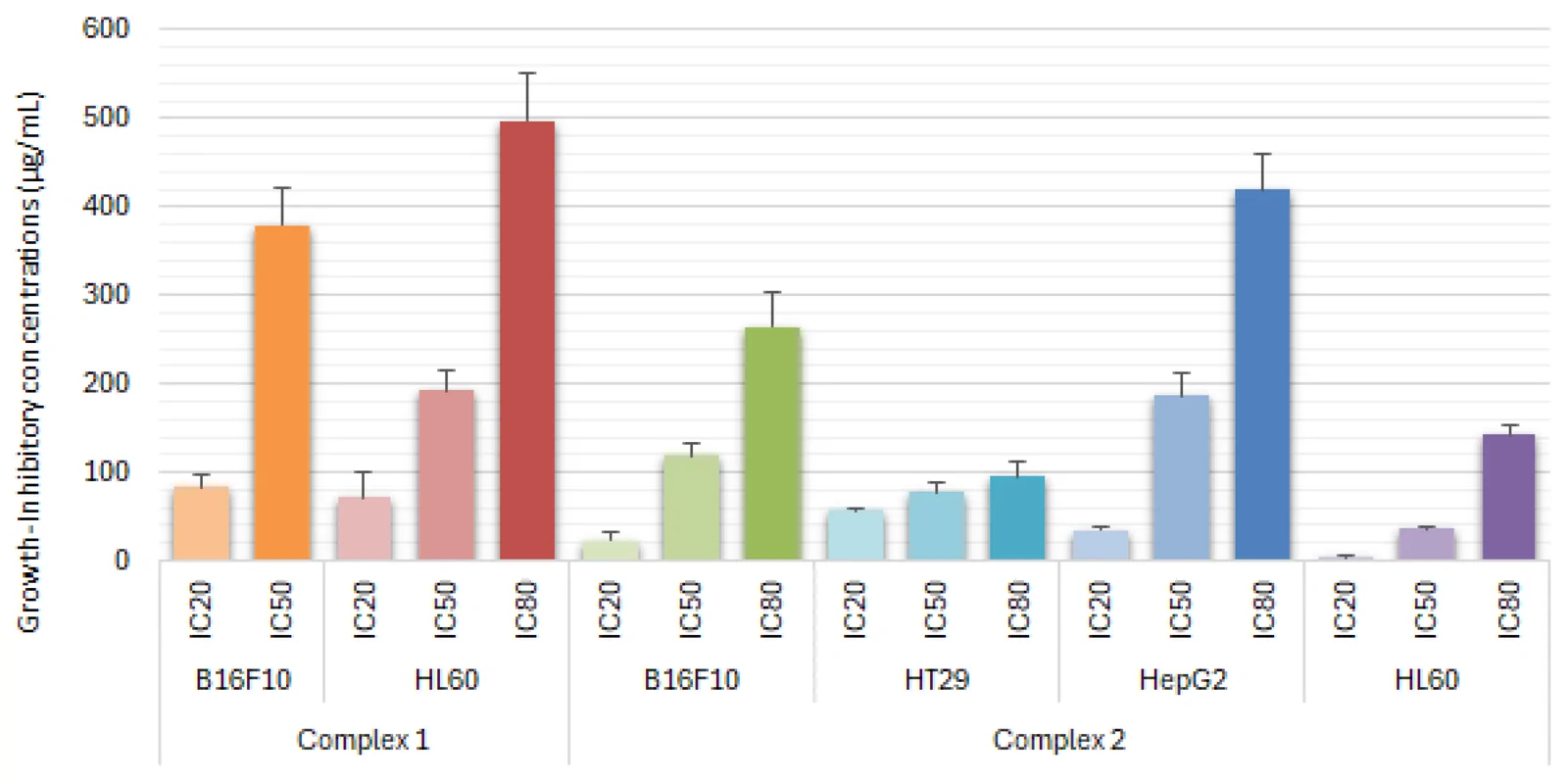
Growth-inhibitory effects of complexes **1** and **2** on HT29, HepG2, B16-F10, and HL-60 cancer cell lines, expressed as IC_20_, IC_50_, and IC_80_ values (μg/mL) after 72 h of treatment. Values represent the mean ± SD of at least two independent experiments performed in triplicate.

Complex **1** did not exhibit measurable cytotoxicity against the HT29 or HepG2 cell lines under any of the tested conditions. Similarly, no significant inhibition of B16-F10 cell proliferation was observed at the IC_80_ level, although minimal effects were detected at lower concentrations, with IC_20_ and IC_50_ values of 82.74 and 378.98 μg/mL, respectively. Complex **1** demonstrated weak cytotoxic activity in the HL-60 cell line, with IC_20_, IC_50,_ and IC_80_ values of 71.97, 193.00, and 497.62 μg/mL, respectively (Figure 12). Overall, these results indicate that complex **1** displayed limited antiproliferative activity in the tumour cell lines evaluated, with the most evident effect observed in HL-60 cells. Its lack of measurable activity in HepG2 cells should be interpreted as low sensitivity of this tumour-derived hepatic cell model under the experimental conditions tested.

**Figure 13:**
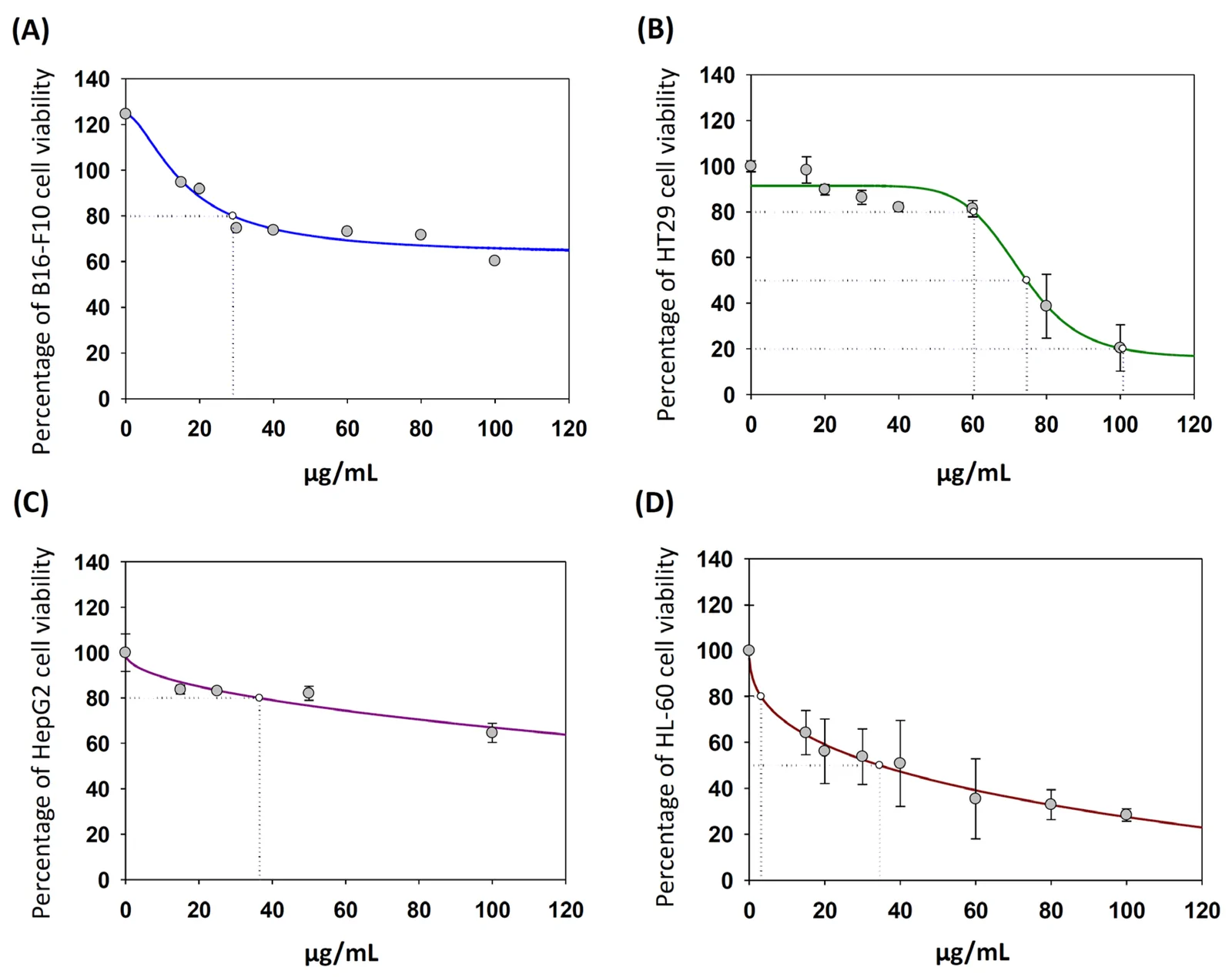
Sigmoidal dose–response curves of complex **2** in (**A**) B16-F10, (**B**) HT29, (**C**) HepG2, and (**D**) HL-60 cells following 72 h of treatment with increasing concentrations (0–100 μg/mL). Each data point represents the mean ± standard deviation (SD) of at least two independent experiments performed in triplicate. Dashed lines indicate the IC_20_, IC_50_, and IC_80_ values, corresponding to the concentrations required to inhibit cell growth by 20%, 50%, and 80%, respectively.

By contrast, complex **2** exhibited more pronounced cytotoxic effects across all the cancer cell lines tested. The lowest activity was observed against HepG2 cells, with IC_20_, IC_50_, and IC_80_ values of 35.39, 185.95, and 419.16 μg/mL, respectively. This lower sensitivity in HepG2 cells, a hepatocellular carcinoma-derived line that retains some hepatic-like features, may be considered a favourable preliminary observation. However, in the absence of non-tumour hepatic or haematopoietic cell models, the present findings cannot be interpreted as direct evidence of low hepatotoxicity, tumour selectivity, or a therapeutic window. Complex **2** demonstrated moderate and comparable antiproliferative effects against B16-F10 and HT29 cells, with IC_50_ values of 119.01 and 77.75 μg/mL, respectively. Notably, complex **2** displayed the highest cytotoxic activity in the HL-60 leukaemia cell line, with an IC_50_ value of 36.98 μg/mL. Furthermore, its IC_20_ value in HL-60 cells (5.78 μg/mL) was the lowest among all cell lines and treatment conditions, suggesting activity at relatively low concentrations. These findings are consistent with the sigmoidal dose–response curves shown in Figure 13. Overall, the marked difference between complexes **1** and **2** supports the relevance of the metal centre in modulating the antiproliferative profile of this hydrogenophosphite framework, with complex **2** showing the most favourable activity pattern, particularly in HL-60 cells.

The DFT study may help to rationalize the different biological profiles observed for the Co(II) complex and the Mg(II) analogue. Although the Mg(II) and Co(II) compounds share the same stoichiometric composition and are isostructural, complex **2** exhibited a markedly stronger cytotoxic effect, particularly against HL-60 cells. This difference may be related, at least in part, to the distinct electronic properties of the metal centres. The Co(II) complex showed a markedly lower HOMO-LUMO energy gap (0.134 eV vs. 2.207 eV), lower chemical hardness, and higher softness and electrophilicity. These descriptors indicate greater electronic reactivity and polarizability for complex **2**, which is consistent with its stronger antiproliferative profile. Complex **1**, formulated as (NH_4_)_2_[Mg(H_2_O)_6_]_3_(HPO_3_)_4_, contains Mg(II), an abundant and generally biocompatible ion with limited redox activity [14], and displayed weak antiproliferative activity across the tested cell lines. In contrast, complex **2**, (NH_4_)_2_[Co(H_2_O)_6_]_3_(HPO_3_)_4_, incorporates Co(II), resulting in an electronic profile distinct from that of the Mg(II) analogue. Although ROS production was not directly measured in the present study, cobalt-containing systems have previously been associated with oxidative stress, mitochondrial dysfunction, apoptosis, and cell-cycle effects in different cancer cell models [15,16,17]. Notably, cobalt- and zinc-based complexes can be fine-tuned using 5-nitropicolinic acid ligands to produce dual anti-inflammatory and anticancer properties, thereby further emphasizing the role of metal identity and ligand environment in modulating bioactivity [18]. Taken together, the higher cytotoxic activity of complex **2** in HL-60 cells may be consistent with a greater capacity of the cobalt-containing framework to perturb cellular homeostasis, although the precise molecular mechanisms require further investigation.

In light of the above results and its higher cytotoxic activity against the HL-60 cell line, complex **2** was selected for further morphological and cytometric analyses. The aim of these studies was to investigate its effects on apoptotic cell populations, cell-cycle distribution, and mitochondrial membrane potential.

Cell morphology. Phase-contrast microscopy was used to evaluate the morphological effects of complex **2** on HL-60 cells after 72 h of treatment. Untreated control cells showed the characteristic rounded and relatively homogeneous appearance of this cell line, with well-defined cell contours and little cellular debris. In contrast, treatment with complex **2** at the IC_50_ concentration produced a more heterogeneous cell population, with a lower proportion of apparently intact cells and an increase in cells displaying altered contours and reduced size.

**Figure 14:**
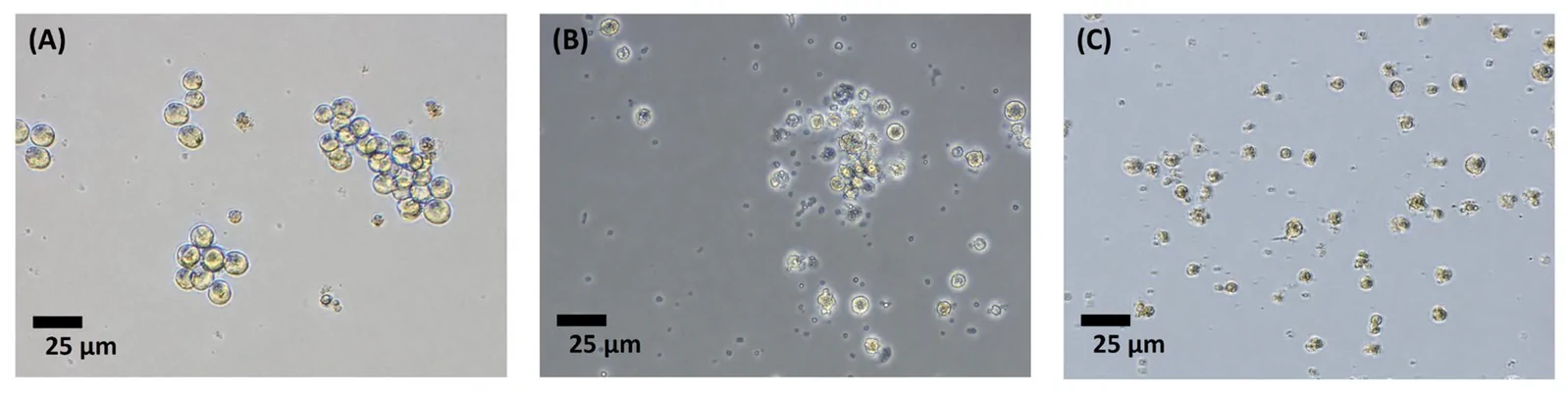
Representative phase-contrast microscopy images of untreated HL-60 cells and cells treated with complex **2** for 72 h at its IC_50_ and IC_80_ concentrations. Images correspond to untreated control cells (**A**), cells treated at the IC_50_ concentration (**B**), and cells treated at the IC_80_ concentration (**C**).

These morphological changes were more pronounced at the IC_80_ concentration, where fewer intact cells and a greater number of cellular debris were observed. Overall, the microscopy images showed a concentration-dependent deterioration of HL-60 cell morphology, in agreement with the reduction in cell viability and the increase in apoptotic cell populations detected in the subsequent Annexin V-FITC/PI assay (Figure 14).

Induction of apoptosis. There are two primary mechanisms of cell death: necrosis and apoptosis. Necrosis is an unregulated process triggered by severe cellular damage, which often results in the lysis of cell populations and the induction of local inflammation [50]. By contrast, apoptosis is a tightly controlled form of programmed cell death characterized by specific morphological and molecular changes, such as chromatin condensation, nuclear fragmentation, membrane blebbing, and the formation of apoptotic bodies [51]. Unlike necrosis, apoptosis generally does not trigger an inflammatory response. A hallmark of early-stage apoptosis is the loss of plasma membrane asymmetry, which is manifested by the translocation of phosphatidylserine (PS) from the inner to the outer leaflet of the cell membrane. This process can be detected by Annexin V binding and was used here to identify apoptotic cell populations by flow cytometry.

**Figure 15:**
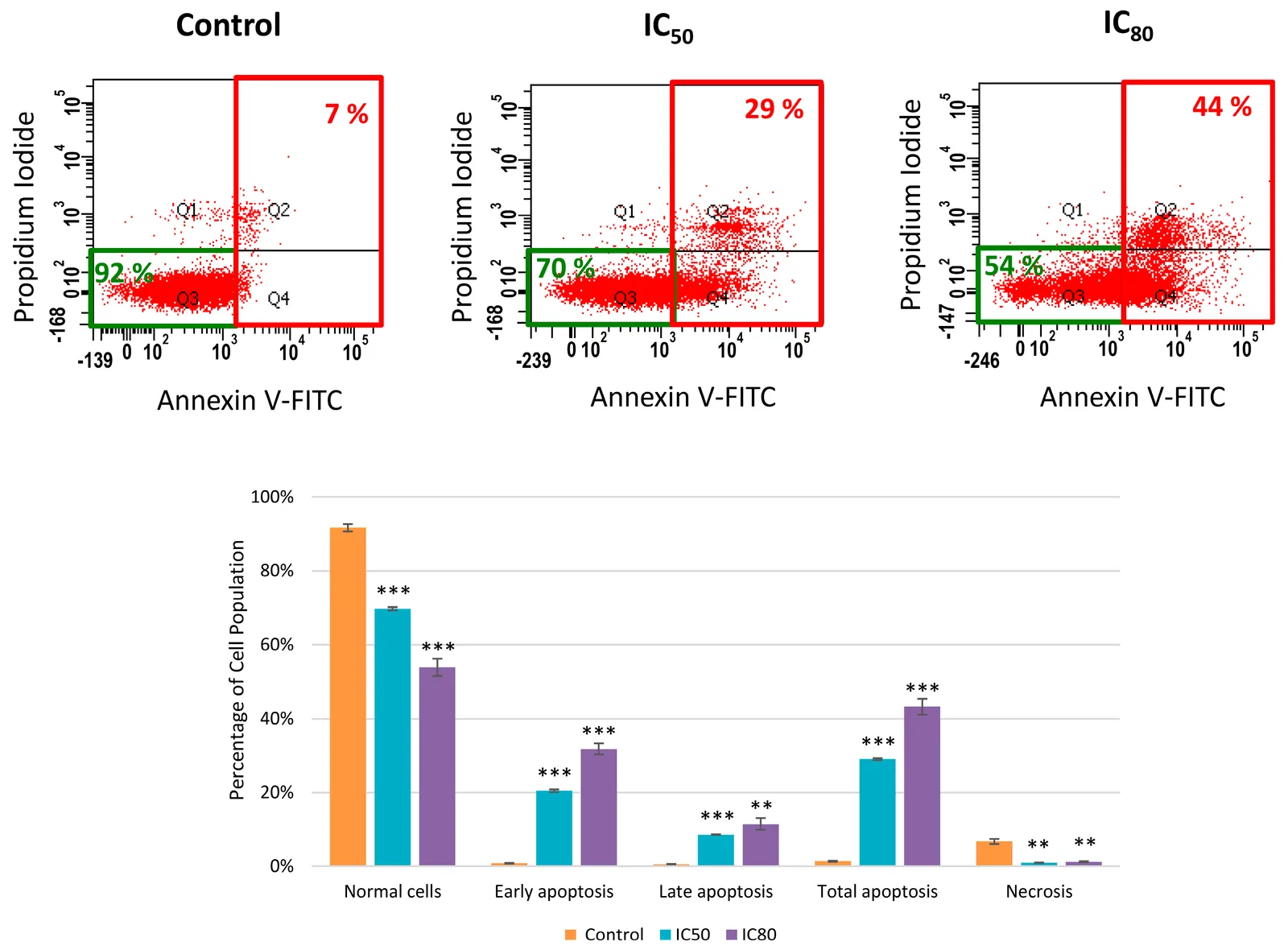
In vitro mechanistic studies of the cytotoxicity of complex **2** in HL-60 cells after 72 h of treatment. (**Top**) Annexin V/PI dot plots: Q1, necrotic cells (Annexin V^−^ PI^+^); Q2, late apoptotic cells (Annexin V^+^ PI^+^); Q3, normal cells (Annexin V^−^ PI^−^); Q4, early apoptotic cells (Annexin V^+^ PI^−^). (**Bottom**) Quantification of cell populations: normal cells (Annexin V^−^ PI^−^), early apoptotic cells (Annexin V^+^ PI^−^), late apoptotic cells (Annexin V^+^ PI^+^), necrotic cells (Annexin V^−^ PI^+^), and total apoptotic cells (early apoptotic + late apoptotic cells). The green and red boxes indicate normal (Annexin V^−^/PI^−^) and total apoptotic (Annexin V^+^) cell populations, respectively; the percentages shown correspond to these populations. Values represent the mean ± SD of two experiments performed in triplicate. Key: *p* ≤ 0.01 (**), and *p* ≤ 0.001 (***), with respect to untreated control cells.

In this context, we investigated whether the cytotoxic effects of complex **2** in HL-60 cells (human acute promyelocytic leukaemia) were associated with the induction of apoptosis. To this end, we performed Annexin V double-staining with fluorescein isothiocyanate (FITC) and propidium iodide (PI), followed by flow cytometric analysis (FACS). Annexin V binds to externalized phosphatidylserine (PS), while propidium iodide (PI) only penetrates cells with compromised membranes and binds to DNA. This dual-staining technique distinguishes four cell populations: viable cells (Annexin V^−^/PI^−^), early apoptotic cells (Annexin V^+^/PI^−^), late apoptotic cells (Annexin V^+^/PI^+^) and necrotic cells (Annexin V^−^/PI^+^). HL-60 cells were analysed 72 h after treatment with complex **2** at the previously determined IC_50_ and IC_80_ concentrations, 36.98 and 142.64 μg/mL, respectively, to assess the concentration-dependent apoptotic response (Figure 15).

Treatment with complex **2** was associated with a dose-dependent increase in Annexin V-positive apoptotic cell populations. In untreated control samples, 92% of cells remained viable. This proportion decreased to 70% and 54% following treatment at IC_50_ and IC_80_, respectively. Total apoptosis rates increased from 2% in control cells to 29% and 44% at IC_50_ and IC_80_, respectively. Notably, early apoptotic cells accounted for the majority of the apoptotic population, representing 21% and 32% of total cells at IC_50_ and IC_80_, respectively. Furthermore, the increase in apoptotic cell populations was more pronounced than the change observed in necrotic cells. The necrotic population remained low after treatment, with values of 0.95% and 1.2% at IC_50_ and IC_80_, respectively, compared with 6.7% in control cells. These results indicate that, under the experimental conditions tested, the cytotoxic effect of complex **2** in HL-60 cells was mainly associated with apoptotic rather than necrotic cell death.

Necrosis is a form of cell death that occurs as a result of pathological conditions, such as physical injury or disease, or an interruption to the blood supply [52]. It is generally considered an unregulated and accidental process that often leads to cell lysis, inflammation, and damage to surrounding tissues. In cancer, necrosis often arises due to adverse microenvironmental factors such as hypoxia, ischaemia, or chronic inflammation. The HL-60 cell line is widely used in leukaemia research to study apoptotic and necrotic pathways. In HL-60 cells, all-trans retinoic acid (ATRA) has been reported to induce differentiation and apoptosis rather than necrotic cell death [53,54]. This observation is consistent with the predominantly apoptotic response observed after treatment with complex **2** in the present study.

In this context, complex **2** was found to have a predominantly pro-apoptotic effect on HL-60 cells, inducing apoptosis while maintaining low levels of necrotic cell death. This shift towards programmed cell death could be a favourable feature, as it may favour the apoptotic elimination of leukaemic cells while limiting necrosis-associated inflammatory responses.

In cancer research, one promising strategy involves using metal-based complexes to trigger apoptosis in cancer cells. While there have been no direct studies evaluating the present magnesium or cobalt hydrogenophosphite complexes specifically in HL-60 leukaemia cells, several reports have demonstrated the pro-apoptotic effects of metal complexes in various cancer cell models. Cobalt-based Schiff base complexes, in particular, have demonstrated marked anti-cancer activity, which has been associated with their ability to interact with DNA, generate reactive oxygen species (ROS) and initiate apoptosis. These complexes often disrupt the mitochondrial membrane potential (ΔΨm), which can lead to caspase activation and programmed cell death. These reported mechanisms support the interest of cobalt complexes as a broader class of anticancer agents [55,56,57].

In parallel, although they have been studied less frequently in the oncological context, magnesium-based coordination complexes have demonstrated notable biocompatibility and biological responsiveness. These complexes have also been reported to show anti-cancer activity associated with DNA interaction and oxidative-stress-related pathways. These findings suggest that Mg^2+^-containing systems may influence cellular responses through context-dependent ionic and coordination interactions [16,58,59].

To date, the anti-cancer potential of hydrogenophosphite-based metal complexes remains largely unexplored, either in isolation or as part of a hybrid metal–organic framework (MOF) system. Nevertheless, metal-phosphonate and related phosphorus-containing coordination systems have attracted growing attention in biomedical research thanks to their structural stability, metal-binding versatility, and inherent biocompatibility. These ligands form robust coordination networks with transition metals, enabling the synthesis of biologically active materials with potential biomedical relevance. Metal-phosphonate nanoparticles have been reported to exhibit efficient cellular uptake and to preserve or deliver biologically functional cargo. Similarly, molecular-level insights into the recognition of phosphite and hypophosphite ligands by biological systems have been provided, supporting the biological relevance of phosphorus-containing coordination environments [60,61].

More broadly, functionalising metal–organic frameworks (MOFs) for apoptosis induction can involve several synergistic strategies. Firstly, the selection of metal ions such as Fe^2+^ or Cu^2+^ has been reported to enable the generation of reactive oxygen species (ROS) via Fenton-like reactions, thereby damaging cellular components and contributing mitochondrial-associated apoptosis [16,55,56,57]. Secondly, modifying the MOF ligands with tumour-targeting groups or pH-sensitive linkers improves the selectivity of drug delivery and enables controlled release in the acidic tumour microenvironment, improving delivery control [8,16]. Thirdly, MOFs can be engineered to co-deliver pro-apoptotic drugs such as doxorubicin or camptothecin. Their porous structure enables high drug loading and sustained release, amplifying the cytotoxic effects and promoting apoptosis in cancer cells [8,16,62].

Cell cycle arrest and distribution. The eukaryotic cell cycle is a precisely regulated series of events governing cell growth, DNA replication, and division. It comprises four sequential phases: G1 (gap 1), S (DNA synthesis) and G2 (gap 2), collectively known as interphase, and M (mitosis). During the G1 phase, cells increase in size and synthesize the proteins and nucleotides required for genome duplication. The S phase then faithfully replicates the entire DNA content. During the G2 phase, cells complete protein synthesis and activate DNA-damage checkpoints to ensure replication accuracy. Finally, the M phase executes mitosis and cytokinesis, segregating the sister chromatids and dividing the cytoplasm to form two genetically identical daughter cells.

Flow cytometry was used to analyse the DNA content of HL-60 cells and to detect changes in the cell-cycle distribution in cells treated with complex **2**, (NH_4_)_2_[Co(H_2_O)_6_]_3_(HPO_3_)_4_. The cells were exposed to its IC_50_ and IC_80_ concentrations for 72 h, after which they were fixed, stained with propidium iodide (PI) and analysed by flow cytometry. DNA histogram analysis enabled the quantification of cell distribution across the G_0_/G_1_, S, and G2/M phases, revealing distinct dose-dependent changes in cell-cycle progression. These results indicate that complex **2** induced cell-cycle arrest in HL-60 cells, which may contribute to its antiproliferative activity.

**Figure 16:**
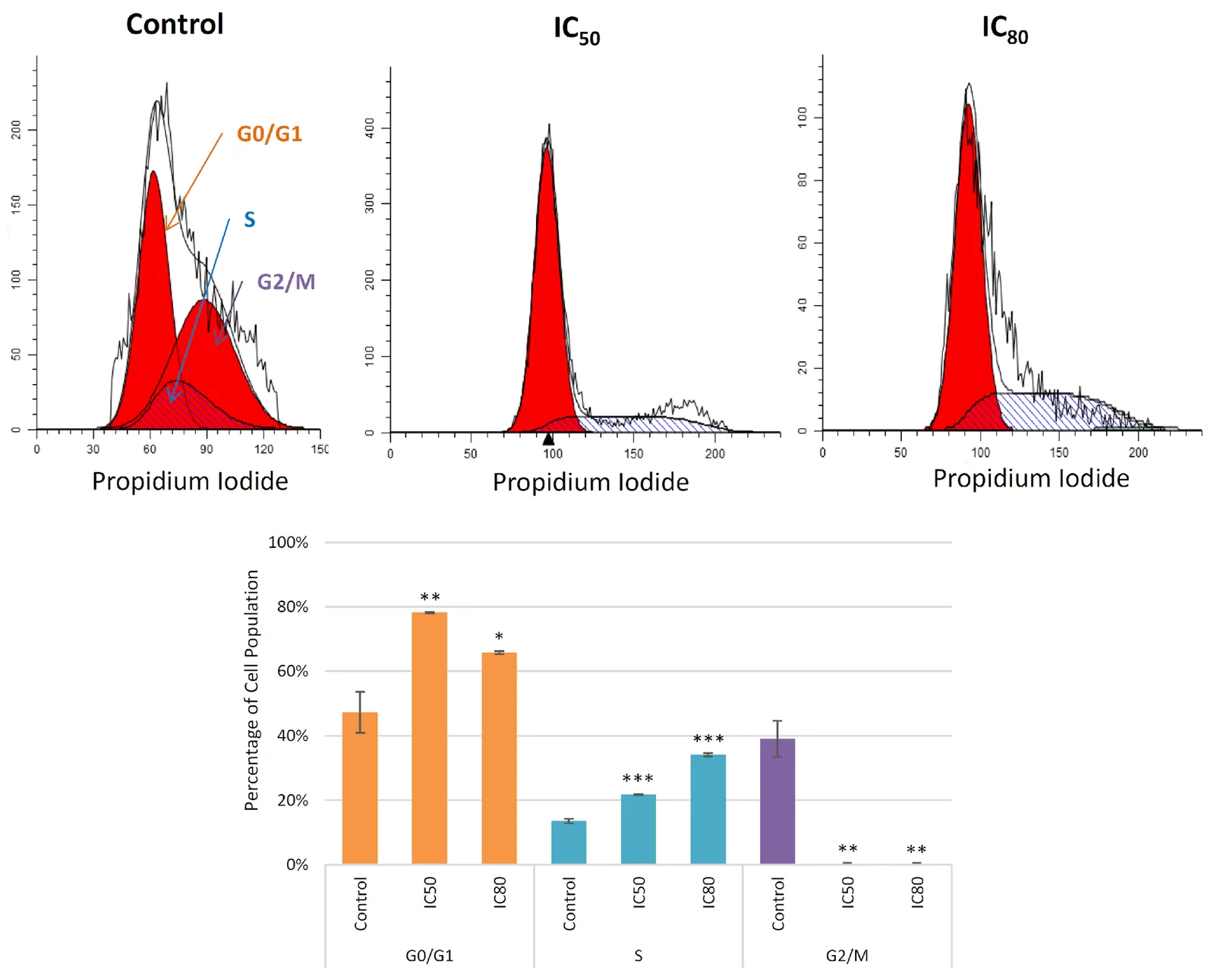
(**Top**): Histograms of cell cycle of HL-60 leukaemia cells, after 72 h of treatment with complex **2**, at IC_50_ and IC_80_ concentrations. G0/G1 phase (orange arrow), S phase (blue arrow), and G2/M phase (purple arrow). (**Bottom**): Percentage of cells in each cell-cycle phase: G0/G1 phase (orange bars), S phase (blue bars), and G2/M phase (purple bars). Control (untreated cells); samples (cells treated with IC_50_ and IC_80_ concentrations of complex **2**). Each value represents mean ± SD of at least two independent experiments performed in triplicate. Key: *p* < 0.05 (*), *p* ≤ 0.01 (**), and *p* ≤ 0.001 (***), with respect to untreated control cells.

DNA histogram analysis (Figure 16) revealed that complex **2** induced significant alterations in HL-60 cell-cycle distribution relative to untreated controls. At IC_50_ and IC_80_, the G_0_/G_1_ population increased to 78.3 ± 0.2% and 65.8 ± 0.5%, representing 1.7- and 1.4-fold increases, respectively. Concurrently, the S-phase fraction increased to 21.8 ± 0.2% (1.6-fold) at IC_50_ and to 34.0 ± 0.5% (2.5-fold) at IC_80_. In contrast, the G2/M compartment decreased from 39.1 ± 5.6% in control cells to undetectable levels (0%) at both concentrations. These results indicate that complex **2** induced cell-cycle arrest involving accumulation in the G0/G1 and S phases, which may contribute to the reduced proliferation observed in HL-60 cells.

Previous studies have indicated that cobalt-based complexes, particularly those coordinated with Schiff base ligands, can trigger cell cycle arrest at the G2/M phase and induce apoptosis in human breast (MCF-7) and lung (A549) cancer cells. These effects have been associated with to the production of reactive oxygen species (ROS) and the disruption of mitochondrial function [63]. Similarly, cobalt (III) complexes with polypyridyl ligands, such as bipyridine or phenanthroline, are known to intercalate DNA and inhibit topoisomerase enzymes, thereby disrupting the cell cycle [64]. Together, these findings support the broader interest of rationally designed cobalt complexes as anticancer agents active in leukaemia models, including HL-60 cells. Recent studies have shown that fine-tuning the ligand architecture in cobalt complexes enhances both anti-cancer and anti-inflammatory activities, highlighting their multifunctional bioactivity [18].

Changes in the mitochondrial membrane potential (MMP). The mitochondrial membrane potential (ΔΨm) is a critical indicator of mitochondrial integrity and is widely used to detect the early stages of intrinsic apoptosis in cancer cells. The collapse of ΔΨm is widely associated with mitochondrial dysfunction during apoptosis and is commonly used as an indicator of mitochondrial involvement in apoptotic cell death. In contrast, apoptotic responses may also be initiated through extrinsic signalling pathways, which do not necessarily require early mitochondrial depolarization. Therefore, monitoring ΔΨm in treated cells can provide insight into the involvement of mitochondrial dysfunction during drug-induced cell death: a significant decrease in ΔΨm supports apoptosis associated with mitochondrial depolarization, whereas preserved ΔΨm may argue against early mitochondrial involvement. This information is useful for clarifying the role of mitochondrial dysfunction in the apoptotic response to novel anticancer compounds [65].

**Figure 17:**
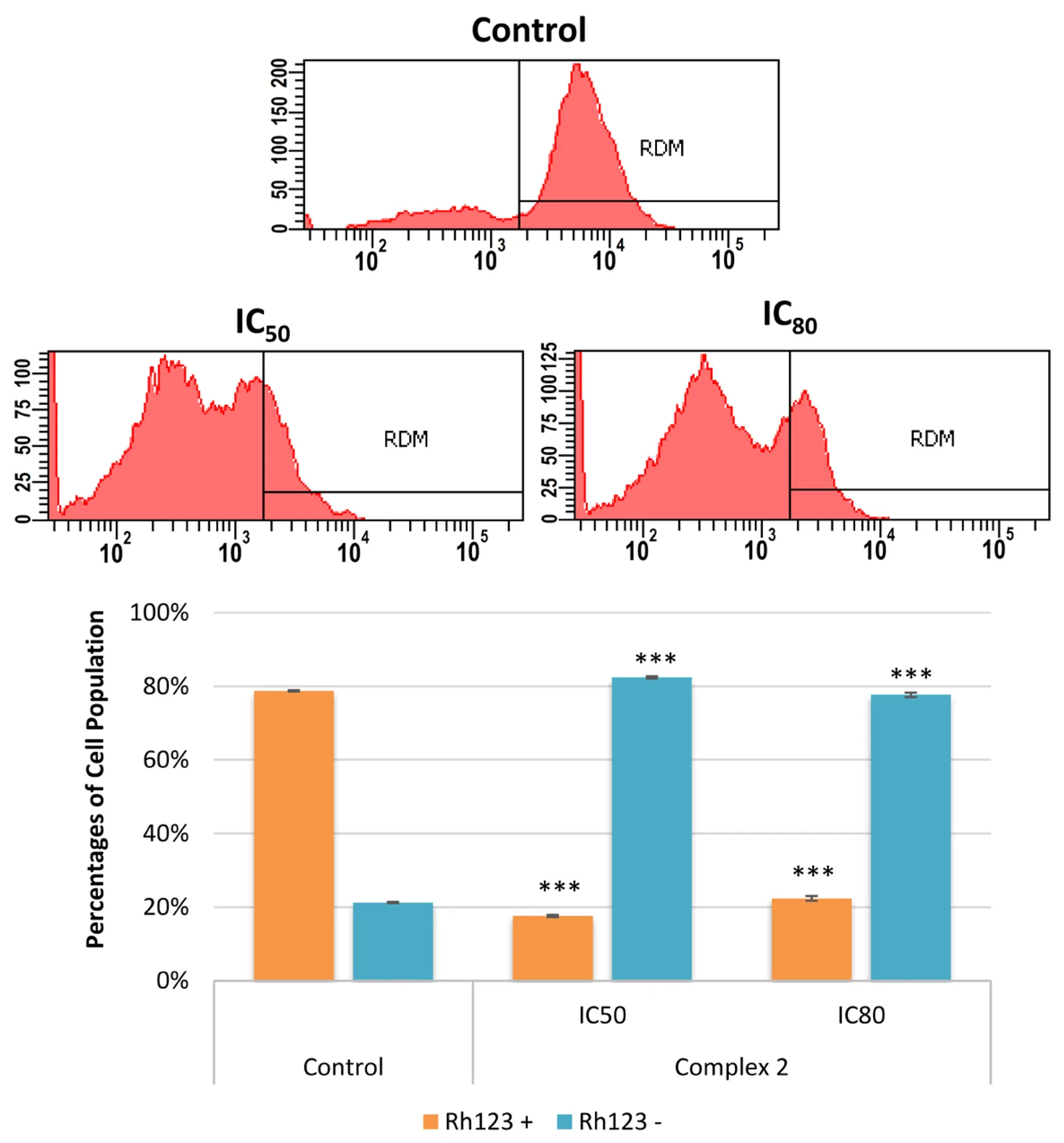
Flow-cytometry analysis of Rh123 and PI staining after exposure of HL-60 cells to complex **2** at its IC_50_ and IC_80_ concentrations for 72 h. Control (untreated cells); samples (cells treated with complex **2**). (**Top**) Diagrams of Rh123 cytometry. The red histograms show the Rh123 fluorescence distributions. The black gating lines delineate the RDM region corresponding to Rh123-positive cells. (**Bottom**) Percentages of HL-60-Rh123-positive and Rh123-negative cells. Values are expressed as the mean ± SD of two independent experiments performed in triplicate. Key: *p* ≤ 0.001 (***), with respect to untreated control cells.

Changes in mitochondrial membrane potential (ΔΨm) were evaluated in HL-60 cells after exposure to complex **2**, (NH_4_)_2_[Co(H_2_O)_6_]_3_(HPO_3_)_4_. MMP integrity was assessed using flow cytometry and dual staining with Rhodamine 123 (Rh123) and propidium iodide (PI). Rh123 is a lipophilic cationic dye that accumulates in energized mitochondria in proportion to ΔΨm, whereas PI only penetrates cells with compromised plasma membranes. Therefore, a decrease in Rh123 fluorescence intensity indicates dissipation of ΔΨm and mitochondrial dysfunction. Compared to untreated controls, treatment with complex **2** at its IC_50_ and IC_80_ concentrations resulted in a significant decrease in the number of Rh123-positive cells (by 78% and 72%, respectively), accompanied by an increase in the number of Rh123-negative (ΔΨm-dissipated) cells. Taken together, the increase in apoptotic cell populations and the concomitant loss of mitochondrial membrane potential indicate apoptosis associated with mitochondrial depolarization, consistent with mitochondrial involvement in the apoptotic response (Figure 17).

The marked mitochondrial dysfunction observed at both concentrations is consistent with apoptotic events involving loss of mitochondrial membrane potential. Taken together with the Annexin V/PI results, this mitochondrial destabilization indicates apoptosis associated with mitochondrial depolarization in HL-60 cells and supports an important pro-apoptotic effect of complex **2** under the experimental conditions tested.

Metal–organic complexes, particularly Co(III) Schiff-base derivatives, have demonstrated marked intrinsic apoptotic activity in cancer cells. These effects have been associated with disruption of mitochondrial membrane integrity, reactive oxygen species (ROS) generation, and caspase activation, which are characteristic features of mitochondrial-associated apoptosis [66].

Organic ligands, such as Schiff bases, phosphonates, and dithiocarbamates, are important for modulating the stability, solubility, and biological activity of metal complexes. Schiff bases may confer DNA-binding affinity and redox activity [55,56,57,63]; phosphonate ligands may enhance biocompatibility and aqueous solubility [4,60]; and dithiocarbamates have been associated with reactive oxygen species (ROS) generation and membrane disruption [67].

Furthermore, the coordination of doxorubicin with metal centres (e.g., Mg^2+^ and Co^2+^) has been demonstrated to increase cytotoxicity and apoptosis in breast cancer models, highlighting the biomedical relevance of metal-coordination strategies in anticancer research [68].

#### 2.4.2. Anti-Inflammatory Activity

RAW 264.7 cell viability. Sub-cytotoxic concentrations of complexes **1** and **2** were determined in RAW 264.7 murine macrophages via the MTT assay across a concentration range of 0–100 μg/mL. This pre-screening step was performed to ensure that subsequent anti-inflammatory evaluations would reflect inhibition of inflammatory mediator production rather than reduced cell viability. Diclofenac (DCF) is a widely used non-steroidal anti-inflammatory drug (NSAID) for the treatment of pain and inflammation associated with conditions such as rheumatoid arthritis, migraines, fever, acute gout, and post-operative traumatic pain. It was included as a reference compound [69]. Following 72 h of incubation, the concentrations required to reduce cell viability by 20, 50 and 80% (IC_20_, IC_50_ and IC_80_, respectively) were determined from the readings of formazan absorbance, providing a complete profile of the cytotoxicity of each complex in RAW 264.7 cells (Table 6).

In RAW 264.7 macrophages, complex **2**, (NH_4_)_2_[Co(H_2_O)_6_]_3_(HPO_3_)_4_, showed the greatest effect on RAW 264.7 cell viability, with an IC_50_ value of 21.42 ± 0.94 μg/mL, approximately three-fold lower than that of the reference drug diclofenac. In contrast, complex **1**, (NH_4_)_2_[Mg(H_2_O)_6_]_3_(HPO_3_)_4_, exhibited reduced cytotoxicity, with an IC_50_ value in the same range as that of diclofenac. These results confirm the need to use compound-specific sub-cytotoxic concentrations for the subsequent NO-inhibition assays.

Nitric oxide (NO) production. Nitric oxide (NO) is widely recognized as a mediator of pro-inflammatory responses and serves as a key biomarker and second messenger for assessing inflammatory activity. It is produced by activated macrophages in response to diverse inflammatory stimuli, including cytokines such as tumour necrosis factor (TNF) and interferon-γ (IFN-γ), bacterial enterotoxins, and lipopolysaccharide (LPS) from Gram-negative bacteria. NO has been implicated in the pathogenesis of various inflammatory conditions, including rheumatoid arthritis, chronic hepatitis, and pulmonary fibrosis [70].

LPS-activated murine RAW 264.7 macrophages are a well-established model for anti-inflammatory screening due to their robust production of nitric oxide (NO), which rapidly oxidises to nitrite. Upon inflammatory stimulation, NO is synthesized by inducible nitric oxide synthase (iNOS), which makes this cell line particularly useful for evaluating the modulation of inflammatory mediators. The Griess reaction is commonly used for the indirect quantification of NO via nitrite determination.

**Figure 18:**
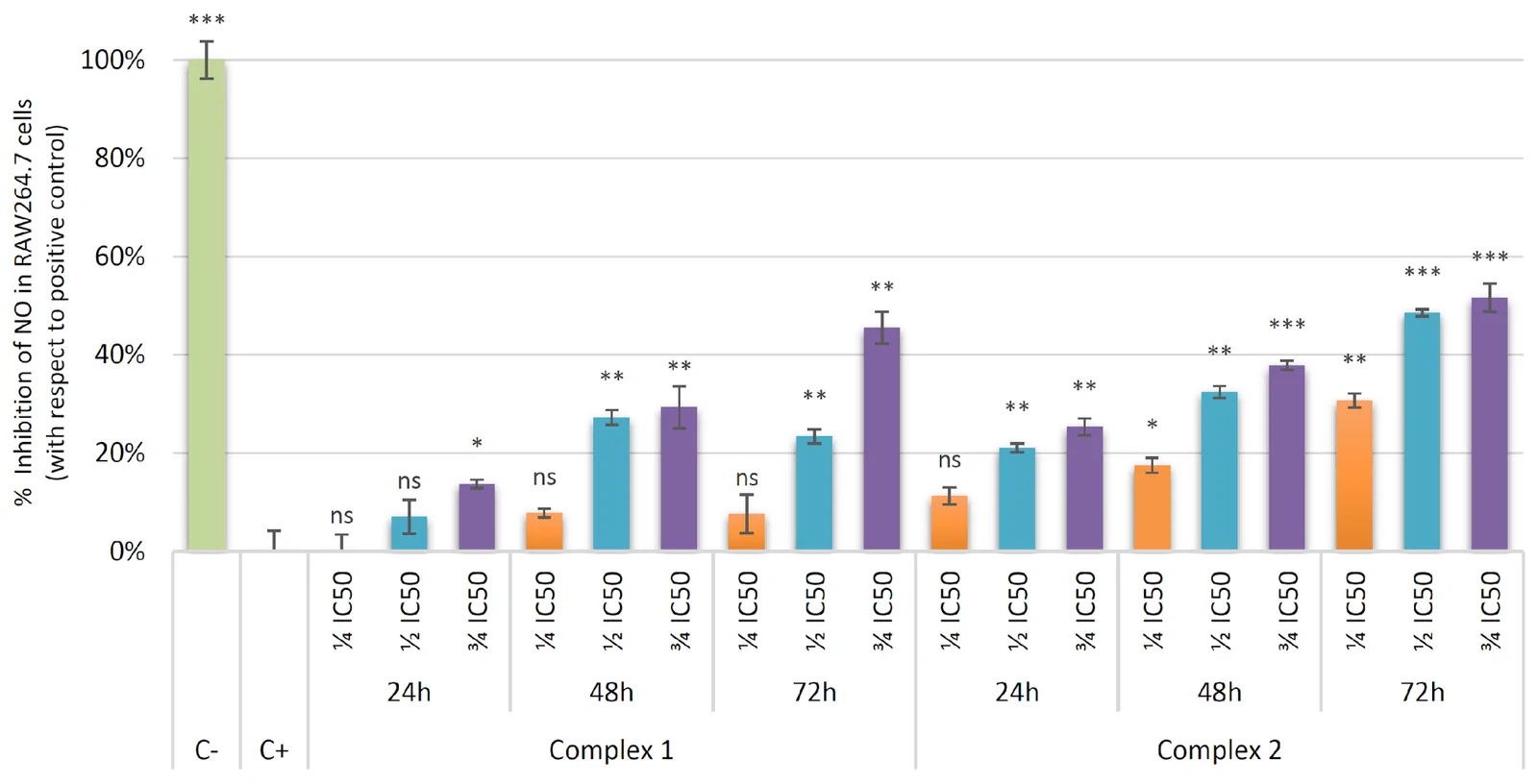
Effect of complexes **1** and **2** on nitrite release in RAW 264.7 murine macrophage cells. Negative control (untreated cells, green); positive control (cells only treated with LPS); and samples (cells treated with LPS and complexes). The complexes were incubated for 24, 48, and 72 h at ¾ IC_50_ (purple), ½ IC_50_ (blue), and ¼ IC_50_ (orange). The data represent the mean ± SD of at least two independent experiments performed in triplicate. Key: ns, not significant; *p* < 0.05 (*), *p* ≤ 0.01 (**), and *p* ≤ 0.001 (***), with respect to the positive control cells.

Anti-inflammatory assays were conducted at sub-cytotoxic concentrations of complexes **1**, **2** and diclofenac: ¼IC_50_, ½IC_50_ and ¾IC_50_. These values, which were derived from previously determined IC_50_ values, are listed in Table 7. They were used to evaluate the inhibitory effects of the complexes on nitric oxide production. Consequently, the NO-inhibitory effects of complexes **1** and **2** were evaluated in LPS-stimulated RAW 264.7 cells by measuring nitrite accumulation after 24, 48 and 72 h of treatment. Both complexes produced concentration-dependent and time-dependent reductions in nitrite levels, with inhibition percentages ranging from 0 to 52% relative to the positive control (Figure 18).

Complex **1**, (NH_4_)_2_[Mg(H_2_O)_6_]_3_(HPO_3_)_4_, exhibited modest, concentration and time-dependent inhibition of nitric oxide (NO) production in lipopolysaccharide (LPS)-stimulated RAW 264.7 macrophages. After 24 h, NO levels decreased by 0%, 7%, and 14% at ¼, ½, and ¾ IC_50_, respectively. The inhibition increased to 8%, 27%, and 29% at 48 h and to 8%, 23%, and 46% at 72 h at these concentrations. These data indicate a moderate NO-inhibitory profile, particularly at the highest concentration tested.

Complex **2**, (NH_4_)_2_[Co(H_2_O)_6_]_3_(HPO_3_)_4_, exhibited markedly stronger NO suppression under the same sub-cytotoxic conditions. After 24 h, NO production decreased by 11%, 21%, and 25% at ¼, ½, and ¾ IC_50_, respectively. By 48 h, inhibition had increased to 18%, 32% and 38% respectively, reaching 31%, 49%, and 52% by 72 h. These results highlight the greater and sustained inhibition of NO production by complex **2** compared with complex **1** under the experimental conditions tested.

**Figure 19:**
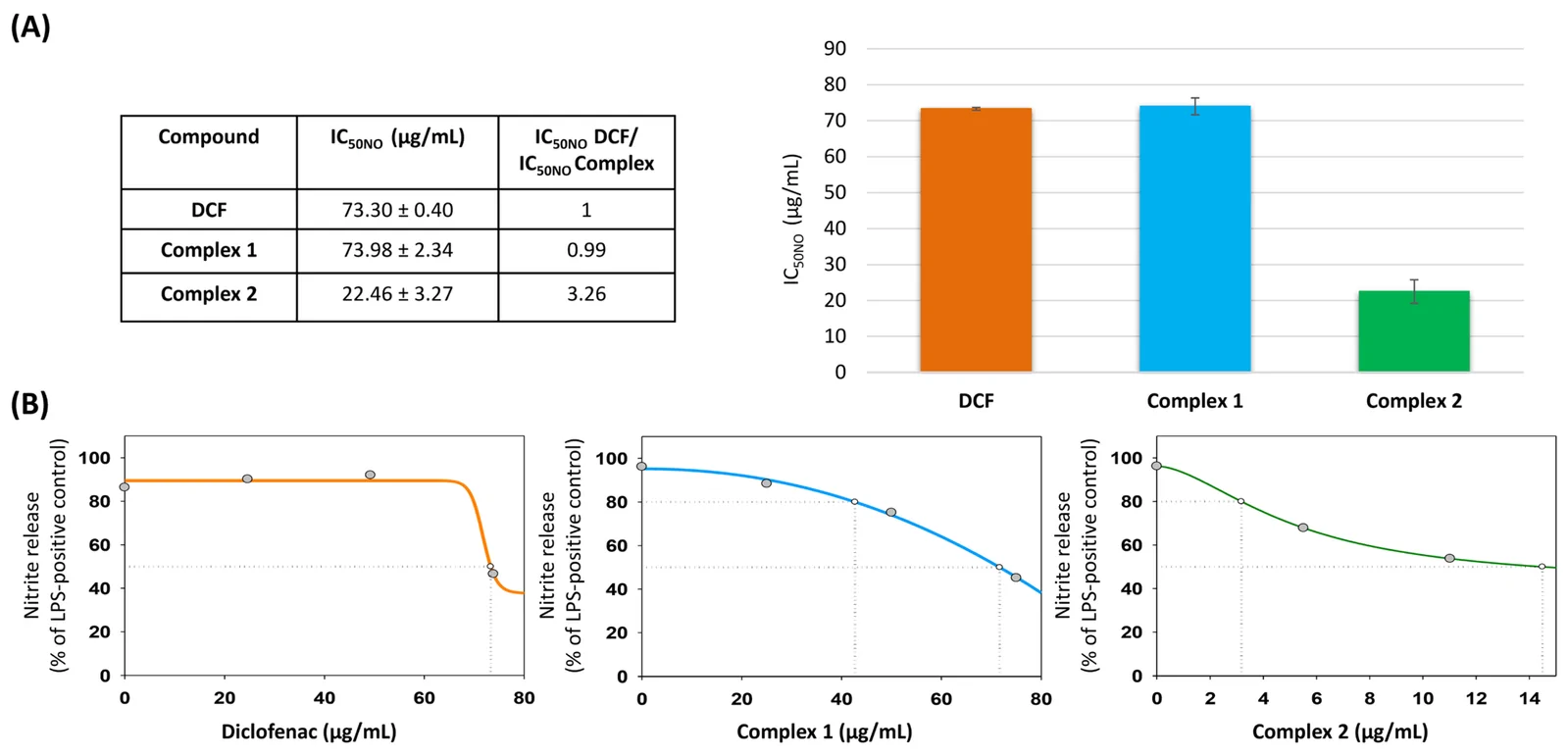
(**A**) NO-release inhibitory effects; IC_50_ NO (μg/mL) concentrations for complexes **1**, **2**, and diclofenac (DCF). (**B**) Sigmoidal curves of the effect of the complexes **1**, **2** and DCF on nitrite release in LPS-activated RAW 264.7 macrophages. The data represent the mean ± SD of at least two independent experiments performed in triplicate.

Concentration of half-maximal inhibition for NO (IC_50_ NO). Although both complexes demonstrated significant inhibition of nitric oxide (NO) release at sub-cytotoxic doses derived from their respective IC_50_ values, this approach complicates direct comparison of their NO-inhibitory activity. To address this issue, we determined the half-maximal inhibitory concentration for NO production (IC_50_ NO) by measuring nitrite accumulation after 72 h of treatment with each complex (Figure 19), since maximal NO inhibition was achieved at this time.

Diclofenac (DCF) was included as a reference control [69]. The results showed that complex **2** was the most active, with an IC_50_ NO value of 22.46 ± 3.27 μg/mL. Under these experimental conditions, this value was approximately three-fold lower than that of diclofenac (IC_50_ NO = 73.30 ± 0.40 μg/mL). However, the IC_50_ NO value of complex **1** (73.98 ± 2.34 μg/mL) was comparable to that of diclofenac, suggesting a similar level of NO-inhibitory activity.

Recent advances in metal–organic frameworks (MOFs) have highlighted their potential relevance in anti-inflammatory applications [71]. The NO-inhibitory effects observed for complexes 1 and 2 are in line with previous reports on Mg-GA-based MOFs, which have been found to reduce inflammatory responses in LPS-stimulated macrophages. In those systems, this effect has been associated with suppression of the NF-κB and MAPK pathways and reduced expression of iNOS and COX-2. Such systems have been proposed for the treatment of bone-related inflammatory disorders [72]. Similarly, cobalt-based MOFs, such as CPO-27(Co) (MOF-74(Co)), have been shown to release nitric oxide in a controlled manner, thereby helping to maintain physiological NO signalling and avoid excessive inflammatory responses [70,71,73].

Although metal phosphite/phosphonate coordination materials incorporating phosphorous acid (H_3_PO_3_) or related phosphorus-containing linkers have been extensively studied for use in catalysis and structural applications, their potential for modulating inflammation has been less explored. This study reports, to our knowledge, the suppression of nitric oxide (NO) production by Co/Mg hydrogenophosphite complexes in LPS-stimulated RAW 264.7 macrophages. The unique coordination chemistry of H_3_PO_3_, which allows versatile bonding m odes with metal centres, offers a potentially useful platform for designing biologically active coordination materials. Given NO’s central role as a pro-inflammatory mediator and the reported immunomodulatory effects of magnesium- and cobalt-based coordination systems, incorporating hydrogenophosphite units into Co/Mg architectures may provide a useful strategy for developing metal-based coordination compounds with NO-inhibitory activity. Accordingly, complexes **1** and **2**, (NH_4_)_2_[Mg(H_2_O)_6_]_3_(HPO_3_)_4_ and (NH_4_)_2_[Co(H_2_O)_6_]_3_(HPO_3_)_4_, exhibited significant NO-inhibitory activity in LPS-stimulated RAW 264.7 macrophages.

## 3. Materials and Methods

### 3.1. Chemistry and Physicochemical Characterisation

#### 3.1.1. Chemicals

Reagent-grade chemicals and solvents were purchased from Sigma-Aldrich (St. Louis, MO, USA) and used as received: MgO (≥99%), H_3_PO_3_ (99%), aqueous ammonia (25%) and CoCl_2_·6H_2_O (98%).

#### 3.1.2. Synthesis

For the magnesium derivative, MgO (55 mg) was dissolved in H_3_PO_3_ (10 mL), followed by the addition of approximately 5 mL of aqueous ammonia solution (0.4 M). The reaction mixture was maintained at 25 °C for 2 h and subsequently kept undisturbed at room temperature. Colourless crystals of (NH_4_)_2_[Mg(H_2_O)_6_]_3_(HPO_3_)_4_ were obtained after one week, isolated by filtration, and washed with an ethanol/water mixture (80:20) [34].

For the cobalt derivative, CoCl_2_·6H_2_O (55 mg) was dissolved in H_3_PO_3_ (10 mL), and 5 mL of aqueous ammonia solution (0.4 M) was then added. The resulting mixture was maintained at 25 °C for 2 h and left to stand at room temperature for one week. Light-pink crystals of (NH_4_)_2_[Co(H_2_O)_6_]_3_(HPO_3_)_4_ were isolated by filtration and washed with an ethanol/water mixture (80:20) [35].

#### 3.1.3. Infrared Measurement

FTIR spectra were acquired between 400 and 4000 cm^−1^ using a JASCO 6200 spectrophotometer (JASCO International Co., Ltd., Hachioji, Tokyo, Japan) fitted with a MIRacle single-reflection diamond ATR accessory (PIKE Technologies, Madison, WI, USA).

#### 3.1.4. X-Ray Powder Diffraction

Powder X-ray diffraction data were collected on an XPERT-PRO Panalytical diffractometer equipped with an ultrafast X’Celerator detector (both from PANalytical B.V., Almelo, The Netherlands). Measurements were performed in Bragg–Brentano geometry using Cu Kα_1_ radiation (λ = 1.5405 Å). Diffractograms were recorded over a 2θ range of 5–75° with a step size of 0.017°.

#### 3.1.5. Thermal Analysis

Thermogravimetric analysis (TGA) was carried out using a TA Instruments SDT-Q600 analyser (TA Instruments, New Castle, DE, USA). Samples were heated from room temperature to 800 °C at a rate of 10 °C·min^−1^.

#### 3.1.6. Density Functional Theory (DFT) Simulation Study

The electronic properties of the two phosphite complexes were examined by density functional theory (DFT) calculations using the GGA functional and the DNP basis set implemented in the DMol3 module of BIOVIA Materials Studio 2020 (Dassault Systèmes BIOVIA, San Diego, CA, USA) [74]. Geometry optimization and electronic-structure calculations were carried out with the DMol3 code. Solvent effects were considered using the Conductor-like Screening Model for Real Solvents (COSMO), applying water as the simulated medium [75]. The remaining computational settings were defined using the “fine” accuracy level available in DMol3. The calculations were used to compare the electronic structures and relative reactivity of complexes 1 and 2 through quantum-chemical descriptors, including HOMO and LUMO energies, the HOMO–LUMO energy gap (Δ*E_gap_*), chemical hardness (*η*), electron affinity (*EA*), ionization potential (*IP*), chemical potential (*μ*), electronegativity (*χ*), softness (*S*), and electrophilicity index (*ω*) [75,76].

Δ*E_gap_* = *E_LUMO_* − *E_HOMO_* = *IP* − *EA* (1)

*IP* = −*E_HOMO_* (2)

*EA* = −*E_LUMO_* (3)

*χ* = (*IP* + *EA*)/2 (4)

*μ* = −*χ* (5)

*η* = (*IP* − *EA*)/2 (6)

*S* = 1/(2*η*) (7)

*ω* = *μ*^2^/(2*η*) (8)

#### 3.1.7. Hirshfeld Surface Studies

Hirshfeld surface (HS) analysis was used to examine intermolecular contacts in the phosphite complexes (NH_4_)_2_[Mg(H_2_O)_6_]_3_(HPO_3_)_4_ (**1**) and (NH_4_)_2_[Co(H_2_O)_6_]_3_(HPO_3_)_4_ (**2**). The corresponding two-dimensional fingerprint plots (FPs) were generated using CrystalExplorer 3.1 (University of Western Australia, Crawley, Australia) [77,78]. The normalised contact distance, *d_norm_*, was calculated from the distances between a surface point and the nearest atoms located inside (*d_i_*) and outside (*d_e_*) the surface, together with the corresponding van der Waals radii. On the *d_norm_* surface, contacts close to the sum of the van der Waals radii appear in white, whereas contacts shorter or longer than this distance are represented in red or blue, respectively [79]. The 2D fingerprint plots provide a quantitative breakdown of the Hirshfeld surface into individual intermolecular contact types and allow their relative contributions to crystal packing to be compared.

### 3.2. Biological Assays

#### 3.2.1. Cell Culture and Compound Preparation

The biological activity of complexes **1** and **2** was assessed using four tumour cell lines: B16-F10 mouse melanoma (ATCC CRL-6475), HT29 human colorectal adenocarcinoma (ECACC 9172201; ATCC HTB-38), HepG2 human hepatocellular carcinoma (ECACC 85011430), and HL-60 human promyelocytic leukaemia (ECACC 98070106; ATCC CCL-240). The adherent cell lines B16-F10, HT29, and HepG2 were maintained in Dulbecco’s Modified Eagle Medium (DMEM) supplemented with 2 mM L-glutamine, 10% heat-inactivated foetal bovine serum (FBS), and 100 U/mL penicillin–100 μg/mL streptomycin. Cultures were incubated at 37 °C in a humidified atmosphere with 5% CO_2_. HL-60 suspension cells were maintained under the same incubation conditions in RPMI-1640 medium supplemented with 10% heat-inactivated FBS.

For anti-inflammatory experiments, RAW 264.7 murine monocyte/macrophage cells (ATCC TIB-71) were cultured in RPMI-1640 medium supplemented with 10% heat-inactivated FBS and gentamicin (0.5 μg/mL), under the incubation conditions described above. Adherent cell lines were used at approximately 80–90% confluence, whereas HL-60 cells were collected during the logarithmic growth phase. All cell lines were supplied by the Cell Bank of the University of Granada (Spain). Stock solutions of complexes **1** and **2** were prepared in dimethyl sulfoxide (DMSO) at 0.5 mg/mL, stored at − 20 °C, and diluted in the appropriate culture medium immediately before treatment.

#### 3.2.2. Cell Viability Assay

The antiproliferative effects of the tested complexes were evaluated in the following cell lines: B16-F10 (mouse melanoma), HT29 (human colorectal adenocarcinoma), HepG2 (human hepatocellular carcinoma), HL-60 (human promyelocytic leukaemia) and RAW 264.7 (murine macrophages). Cell viability was determined using MTT or resazurin-based assays according to the cell model analysed. The MTT assay (Sigma-Aldrich, St. Louis, MO, USA) was used for B16-F10, HT29, HepG2, and RAW 264.7 cells. Cells were plated in 96-well plates at densities of 5 × 10^3^ cells/well for B16-F10, 6 × 10^3^ cells/well for HT29, 15 × 10^3^ cells/well for HepG2, and 6 × 10^3^ cells/well for RAW 264.7. After seeding, cells were exposed to complexes 1 and 2 over a concentration range of 0–100 μg/mL for 72 h. MTT solution (100 μL; 0.5 mg/mL, prepared in PBS/medium, 1:1) was then added to each well, and plates were incubated for 1.5 h. The resulting formazan crystals were dissolved in 100 μL of dimethyl sulfoxide (DMSO), and absorbance was measured at 570 nm using a Tecan Sunrise MR20–301 microplate reader (TECAN, Grödig, Austria). All treatments were performed in triplicate in at least two independent experiments.

For HL-60 cells, cell viability was evaluated using the resazurin assay, based on the conversion of resazurin into fluorescent resorufin by metabolically active cells. HL-60 cells (2 × 10^5^ cells/well) were exposed to complexes **1** and **2** at concentrations between 0 and 100 μg/mL for 72 h. After treatment, 20 μL of 1 mM resazurin solution was added to each well, and cells were incubated for an additional 2 h. Resorufin was then solubilised by adding 50 μL of 3% SDS. Fluorescence was recorded at 535 nm excitation and 590 nm emission using a Synergy HTX multi-mode reader (BioTek, Winooski, VT, USA). Cell viability was calculated relative to untreated control cells, which were considered as 100% viability, and results are expressed as the mean ± standard deviation from at least two independent experiments performed in triplicate. IC_20_, IC_50_, and IC_80_ values were defined as the concentrations producing 20%, 50%, and 80% inhibition of cell viability, respectively. Complexes showing relevant cytotoxicity in cancer cell lines were selected for further assays, including apoptosis analysis, cell-cycle evaluation, and mitochondrial membrane potential assessment.

After 72 h of treatment, representative images of untreated HL-60 cells and cells exposed to complex **2** at its IC_50_ and IC_80_ concentrations were acquired using an inverted phase-contrast microscope.

#### 3.2.3. Apoptosis Assay

Apoptosis in HL-60 cells was evaluated by annexin V-FITC/propidium iodide (PI) double staining and subsequent flow-cytometric analysis using a FACS flow cytometer (Coulter Corporation, Hialeah, FL, USA). Cells were seeded in 24-well plates at 5 × 10^5^ cells/well in 1.5 mL of culture medium and allowed to equilibrate for 24 h. They were then exposed, in triplicate, to the selected complex at its previously established IC_50_ and IC_80_ concentrations for 72 h. After treatment, cells were collected and resuspended in binding buffer containing 10 mM HEPES/NaOH (pH 7.4), 140 mM NaCl, and 2.5 mM CaCl_2_. Annexin V-FITC conjugate was added at 1 μg/mL, and samples were incubated for 15 min at room temperature protected from light. Immediately before acquisition, cells were stained with 5 μL of PI solution (1 mg/mL). Approximately 10,000 cells were acquired per experiment, and the assay was carried out twice to confirm reproducibility.

#### 3.2.4. Cell Cycle Assay

Cell-cycle distribution in HL-60 cells was analysed by flow cytometry after propidium iodide (PI) staining of cellular DNA. Since PI fluorescence intensity reflects DNA content, this approach allowed the relative proportions of cells in the G0/G1, S, and G2/M phases to be determined. Data were acquired using an EPICS XL cytometer (Coulter Corporation, Hialeah, FL, USA) with excitation at 488 nm. HL-60 cells were plated in 24-well plates at 5 × 10^5^ cells/well in 1.5 mL of culture medium and pre-incubated for 24 h. Cells were then treated, in triplicate, with complex **2** at its previously determined IC_50_ and IC_80_ concentrations for 72 h. After treatment, cells were washed twice with PBS, collected by gentle centrifugation because they grow in suspension, and resuspended in 1× TBS (10 mM Tris, 150 mM NaCl). DNA staining was carried out using Vindelov buffer (100 mM Tris, 100 mM NaCl, RNase 10 mg/mL, PI 1 mg/mL; pH 8.0) before flow-cytometric acquisition. A minimum of 1 × 10^4^ events was recorded for each sample, and doublets were excluded by pulse processing. Cell-cycle phase distribution was calculated from DNA-content histograms.

#### 3.2.5. Mitochondrial-Membrane Potential (MMP) Assay

Mitochondrial membrane potential (ΔΨm) in HL-60 cells was analysed by flow cytometry using dihydrorhodamine 123 (DHR 123). After intracellular oxidation, non-fluorescent DHR 123 is converted into rhodamine 123 (Rh123), a membrane-permeable cationic dye that preferentially accumulates in polarised mitochondria. Therefore, Rh123 fluorescence intensity reflects ΔΨm, with reduced fluorescence indicating mitochondrial depolarisation and higher fluorescence indicating preserved mitochondrial polarisation. Fluorescence was detected in the FL1 channel using 488 nm excitation and 530 ± 15 nm emission. HL-60 cells were seeded in 24-well plates at 5 × 10^5^ cells/well in 1.5 mL of culture medium and pre-incubated for 24 h. Cells were then treated, in triplicate, with complex **2** at its IC_50_ and IC_80_ concentrations for 72 h. After treatment, culture medium was replaced with fresh medium containing DHR 123 at a final concentration of 5 μg/mL, and cells were incubated for 1 h at 37 °C in 5% CO_2_. Cells were subsequently washed and resuspended in PBS containing propidium iodide (PI, 5 μg/mL) to exclude non-viable cells before analysis on a FACScan cytometer (Coulter Corporation, Hialeah, FL, USA). A minimum of 10,000 events was acquired for each sample, and doublets were excluded by pulse processing.

#### 3.2.6. Griess Assay

RAW 264.7 macrophages were employed as an in vitro model of LPS-induced inflammatory activation. Cells were plated in 24-well plates at 6 × 10^4^ cells/well and stimulated with lipopolysaccharide (LPS, 10 μg/mL) for 24 h. Subsequently, cells were exposed to complexes **1** and **2** or diclofenac at concentrations corresponding to ¾ IC_50_, ½ IC_50_, and ¼ IC_50_, previously determined for each compound, for 24, 48, and 72 h. Culture supernatants were collected, or stored at − 80 °C until analysis, and nitrite accumulation was quantified using the Griess reaction. For this assay, 150 μL of each supernatant or sodium nitrite standard (0–120 μM) was transferred to a 96-well plate and mixed with 25 μL of Griess reagent A (0.1% N-(1-naphthyl) ethylenediamine dihydrochloride) and 25 μL of Griess reagent B (1% sulfanilamide in 5% phosphoric acid). After 15 min at room temperature, absorbance was recorded at 540 nm using a Tecan Sunrise MR20–301 microplate reader (TECAN, Grödig, Austria). Nitrite concentrations were obtained from the sodium nitrite standard curve. NO production was estimated from nitrite accumulation and normalised to the corresponding controls, with untreated cells defined as 0% NO production and LPS-stimulated cells defined as 100%.

#### 3.2.7. Statistical Analysis

Dose–response curves from the cytotoxicity and nitric oxide assays were fitted by nonlinear regression using a four-parameter logistic model in SigmaPlot^®^ 12.5 (Systat Software, San Jose, CA, USA): y = y_min + (y_max − y_min)/[1 + (x/EC50)^-Hill slope^]. The IC_20_, IC_50_, and IC_80_ values were obtained from the fitted curves and correspond to the concentrations producing 20%, 50%, and 80% inhibition, respectively. Quantitative results are presented as the mean ± standard deviation (SD) from at least two independent experiments, each performed in triplicate. Statistical significance was evaluated using Student’s *t*-test, with significance levels indicated in the corresponding figures and tables as *p* < 0.05 (*), *p* ≤ 0.01 (**), and p ≤ 0.001 (***). For each assay, comparisons were performed against the appropriate control, including untreated negative controls or LPS-stimulated positive controls, as applicable.

## 4. Conclusions

In this study, two hybrid phosphite complexes, (NH_4_)_2_[M(H_2_O)_6_]_3_(HPO_3_)_4_ (M = Co, Mg), have been synthesized in powder form at room temperature using wet chemistry, providing a room-temperature synthetic route as an alternative to traditional high-temperature methods. Structural analysis indicated that these complexes are isostructural and possess a similar three-dimensional network, constructed from M(H_2_O)_6_ octahedra (M = Co, Mg), [HPO_3_]^2−^ and [NH_4_]^+^ units that are stabilized by an intricate network of hydrogen bonds. Infrared spectroscopy confirmed the structural data, with the presence of the characteristic vibrational bands of the hydrogenophosphite oxyanion (HPO_3_)^2−^, ammonium cation, and the water molecules. The thermal behaviour of complexes (NH_4_)_2_[Mg(H_2_O)_6_]_3_(HPO_3_)_4_ (**1**) and (NH_4_)_2_[Co(H_2_O)_6_]_3_(HPO_3_)_4_ (**2**) reveals distinct decomposition pathways despite their structural similarities. Complex **1** exhibits an initial degradation above 112 °C, mainly associated with the loss of fifteen water molecules. In contrast, complex **2** undergoes a more continuous thermal decomposition process between 200 and 600 °C. The first stage corresponds to the removal of coordinated water molecules. Overall, complex **1** shows a more stepwise dehydration process, whereas complex **2** displays a more progressive and chemically complex thermal decomposition behaviour. The Hirshfeld surface analysis, along with 2D fingerprint plots, highlighted the important role of hydrogen bonding (O–H∙∙∙O and N–H∙∙∙O) in stabilizing and maintaining the cohesion of the supramolecular structure. These interactions were quantified, highlighting their contribution to crystal packing and structural stability. The two isostructural ammonium hydrogenophosphite complexes, based on Mg(II) and Co(II), were evaluated for biological activities. The cobalt complex showed stronger anticancer effects than its magnesium analogue, inducing apoptosis and mitochondrial depolarization in HL-60 cells. Both complexes also reduced LPS-induced nitric oxide production in macrophages, with complex **2** showing a lower IC_50_ NO value than diclofenac and complex **1** displaying a comparable effect under the experimental conditions tested. To rationalize these findings, density functional theory (DFT) calculations were performed to investigate the electronic properties governing the reactivity of both complexes. The LUMO distributions are mainly localized on Co(H_2_O) and HPO_3_ groups, indicating favourable electron-accepting sites that may be relevant to the relative reactivity of the complexes. The Co(II) complex (NH_4_)_2_[Co(H_2_O)_6_]_3_(HPO_3_)_4_ was found to possess a markedly lower HOMO-LUMO energy gap (0.134 eV), lower chemical hardness, and higher softness and electrophilicity compared with the Mg(II) complex (NH_4_)_2_[Mg(H_2_O)_6_]_3_(HPO_3_)_4_. DFT results support a possible relationship between the stronger biological profile of the Co(II) complex and its higher electronic reactivity and greater polarizability. These results highlight the interest of cobalt-based hydrogenophosphite complexes as bioactive coordination compounds with combined anticancer and NO-inhibitory properties. Overall, this work provides insights into how metal identity and electronic properties can influence the biological response of hydrogenophosphite-based coordination complexes. The behaviour of these complexes under physiologically relevant conditions, including their stability and speciation in biological media, and the proposed thermal decomposition products remain to be established. These insights may contribute to the rational design of new metal-based bioactive coordination compounds, although further studies will be required to define their selectivity, precise molecular mechanisms, and pharmacological relevance.

## Figures and Tables

**Table 1:** Hydrogen bond geometry (Å) of (NH_4_)_2_[Mg(H_2_O)_6_]_3_(HPO_3_)_4_.

D–H∙∙∙A	D–H	H∙∙∙A	D∙∙∙A	D–H∙∙∙A
O8–H81∙∙∙O3	0.751(19)	1.983(19)	2.7227(12)	168.2(18)
O8–H82∙∙∙O1	0.766(17)	1.927(18)	2.6883(12)	172.5(18)
O10–H100∙∙∙O1	0.848(17)	1.833(18)	2.6707(10)	169.2(16)
N1–H110∙∙∙O3	0.90(3)	1.90(3)	2.799(2)	179.(2)
O5–H5∙∙∙O4 ^i^	0.874(17)	1.812(17)	2.6758(10)	169.0(16)
O6–H61∙∙∙O4 ^v^	0.798(19)	1.938(19)	2.7193(12)	166.0(17)
O6–H62∙∙∙O4 ^vi^	0.753(19)	1.999(19)	2.7436(12)	170.3(19)
O7–H71∙∙∙O5 ^vii^	0.71(3)	2.23(3)	2.9363(19)	172.(3)
O7–H72∙∙∙O2 ^vii^	0.87(3)	1.80(3)	2.6657(18)	172.(2)
O9–H91∙∙∙O2 ^iv^	0.85(2)	1.87(2)	2.7207(12)	173.0(17)
N1–H112∙∙∙O8 ^viii^	0.87(2)	2.20(2)	3.0242(17)	157.3(17)

Symmetry codes: (i) x, −y, z; (iv) −x, y, −z; (v) x + $\frac{1}{2}$, y − $\frac{1}{2}$, −z + 1; (vi) x, −y, z + 1; (vii) x, y, z + 1; and (viii) x, −y + 1, z − 1.

**Table 2:** Hydrogen-bond geometry (Å) of (NH_4_)_2_[Co(H_2_O)_6_]_3_(HPO_3_)_4_.

D–H∙∙∙A	D–H	H∙∙∙A	D∙∙∙A	D–H∙∙∙A
O5–H5∙∙∙O4 ^i^	0.807(15)	1.887(15)	2.6771(15)	165.70(18)
O6–H61∙∙∙O4 ^v^	0.813(13)	1.923(13)	2.7306(16)	173.(2)
O6–H62∙∙∙O4 ^vi^	0.805(12)	1.931(12)	2.7279(17)	170.(2)
O7–H71∙∙∙O5 ^vii^	0.81(3)	2.08(3)	2.887(2)	178.(3)
O7–H72∙∙∙O2 ^vii^	0.816(14)	1.866(14)	2.682(2)	180.(4)
O8–H81∙∙∙O3	0.814(18)	1.915(17)	2.7249(17)	173.0(18)
O8–H82∙∙∙O1	0.820(15)	1.875(14)	2.6890(16)	171.4(18)
O9–H91∙∙∙O2 ^iv^	0.817(17)	1.895(17)	2.7102(16)	174.81(19)
O9–H92∙∙∙O1 ^viii^	0.804(12)	1.949(14)	2.7319(16)	165.(2)
O10–H100∙∙∙O1	0.810(17)	1.854(17)	2.6624(15)	175.(2)
N1–H110∙∙∙O3	0.87(2)	1.95(2)	2.813(3)	176.(3)
N1–H112∙∙∙O8 ^ix^	0.866(17)	2.161(17)	3.006(2)	164.9(16)
N1–H113∙∙∙O10	0.87(2)	2.32(2)	3.182(3)	173.(3)

Symmetry codes: (i) x, −y, z; (iv) −x, y, −z; (v) x + $\frac{1}{2}$, y − $\frac{1}{2}$, −z + 1; (vi) x, −y, z + 1; (vii) x, y, z + 1; (viii) −x, y, −z + 1; and (ix) x, −y + 1, z − 1.

**Table 3:** Infrared absorption bands and frequencies assigned to the different vibrations of (NH_4_)_2_[M(H_2_O)_6_]_3_(HPO_3_)_4_ [M = Mg, Co].

Frequencies of the Bands of (NH_4_)_2_[M(H_2_O)_6_]_3_(HPO_3_)_4_ [M = Mg, Co] in cm^−1^		
Complex 1	Complex 2	Attributions
3431	3395	υ_s_ (OH)
3111	2969	υ_s_(NH_4_)
2432	2435	υ_s_(P–H)
1688	1682	δ(OH) of H_2_O
1435	1432	δ(NH_4_)
1069	1052	υ_s_(P=O)
1059	1029	δ(P–H)
975	975	υ_as_(P–O)
860	880	υ_s_(P–O)
573	626	δ_as_(PO_3_)
469	564	δ_s_(PO_3_)

**Table 4:** Quantum-chemical parameters of (NH_4_)_2_[Mg(H_2_O)_6_]_3_(HPO_3_)_4_ and (NH_4_)_2_[Co(H_2_O)_6_]_3_(HPO_3_)_4_ determined using the DFT/GGA theoretical model.

Parameters	Complex 1 (Mg)	Complex 2 (Co)
*E*_LUMO_ (eV)	−2.808	−6.511
*E*_HOMO_ (eV)	−5.016	−6.645
*E*_gap_ (eV)	2.207	0.134
Ionization energy, *I* = −*E*_HOMO_ (eV)	5.016	6.645
Electron affinity, *A* = −*E*_LUMO_ (eV)	2.808	6.511
Chemical hardness, *η* = (*I* − *A*)/2 (eV)	1.104	0.067
Chemical potential, *µ* = − (*I* + *A*)/2 (eV)	−3.912	−6.578
Softness, *S* = 1/(2*η*) (eV^−1^)	0.453	7.485
Electrophilicity index, ω = *µ*^2^/2*η* (eV)	6.932	323.875

**Table 5:** Growth-inhibitory effects of complexes **1** and **2** on the viability of the cancer cell lines HT29, HepG2, B16-F10, and HL-60. The cells were treated with increasing concentrations of each complex for 72 h. The data represent the mean values ± standard deviation (SD) from at least two independent experiments performed in triplicate.

Complex	Cell Line	IC_20_ (μg/mL)	IC_50_ (μg/mL)	IC_80_ (μg/mL)
**Complex 1**	B16-F10	82.74 ± 14.88	378.98 ± 43.39	—
	HL-60	71.97 ± 29.19	193.00 ± 23.37	497.62 ± 54.88
**Complex 2**	B16-F10	23.24 ± 8.19	119.01 ± 14.26	264.88 ± 37.77
	HT29	56.31 ± 2.99	77.75 ± 10.48	95.18 ± 17.10
	HepG2	35.39 ± 1.59	185.95 ± 25.77	419.16 ± 39.74
	HL-60	5.78 ± 0.68	36.98 ± 2.11	142.64 ± 11.54

**Table 6:** Growth-inhibitory effects (IC_20_, IC_50_, and IC_80_, μg/mL) of complexes **1** & **2** and the reference diclofenac (DCF), in RAW 264.7 murine macrophage cells after 72 h of treatment.

Complex	IC_20_ (µg/mL)	IC_50_ (µg/mL)	IC_80_ (µg/mL)	IC_50_ DCF/IC_50_comp#
**DCF**	51.91 ± 0.89	69.00 ± 1.13	90.52 ± 1.85	1
**Complex 1**	69.68 ± 7.99	99.86 ± 4.89	180.85 ± 2.12	0.69
**Complex 2**	17.60 ± 2.99	21.42 ± 0.94	27.25 ± 1.39	3.22

**Table 7:** Sub-cytotoxic concentrations of tested compounds **1**, **2** and diclofenac (DCF) against RAW 264.7 monocyte/macrophage murine cells after 72 h of treatment.

Complex	¼ IC_50_ (µg/mL)	½ IC_50_ (µg/mL)	¾ IC_50_ (µg/mL)
**DCF**	17.25 ± 0.28	34.5 ± 0.56	51.75 ± 0.84
**Complex 1**	24.96 ± 1.22	49.93 ± 2.44	74.89 ± 3.66
**Complex 2**	5.35 ± 0.23	10.71 ± 0.47	16.06 ± 0.70

## Data Availability

The original contributions presented in this study are included in the article. Further inquiries can be directed to the corresponding authors.
